# MMP14 from BM-MSCs facilitates progression and Ara-C resistance in acute myeloid leukemia via the JAK/STAT pathway

**DOI:** 10.1186/s40164-025-00635-6

**Published:** 2025-03-22

**Authors:** Jinxian Wu, Xinqi Li, Yin Liu, Guopeng Chen, Ruihang Li, Hongqiang Jiang, Wanyue Yin, Xiqin Tong, Rui Cao, Xianwang Wang, Xiaoyan Liu, Fuling Zhou

**Affiliations:** 1 Department of Hematology, Zhongnan Hospital of Wuhan University, 169 Donghu Road, Wuhan, 430072 P. R. China; 2 Research Center for Lifespan Health, Wuhan University, Wuhan, 430072 Hubei China; 3 Department of Biochemistry and Molecular Biology, Center for Molecular Medicine, Health Science Center, Yangtze University, Jingzhou, 434023 Hubei China; 4 Shannan Maternal and Child Health Hospital, Shannan, Xizang, 856100 China

**Keywords:** Acute myeloid leukemia, Mesenchymal stromal cells, MMP14, JAK/STAT pathway, PGE2

## Abstract

Growing evidence underscores the pivotal impact of crosstalk between leukemic stem cells (LSCs) and mesenchymal stromal cells (MSCs) within their niche on leukemia initiation, progression, and therapy response. Although MMP14 plays an important role in inflammation and cancer, the regulation and role of MSC-derived MMP14 in acute myeloid leukemia (AML) are largely unknown. Here, we found that AML patient-derived MSCs (AML-MSCs) were more supportive of AML cell growth compared to healthy donor-derived MSCs (HD-MSCs). Moreover, AML-MSCs and HD-MSCs showed significant differences in gene expression and protein expression profiles. Knockdown of MMP14 in MSCs inhibited the CFU-F ability of MSC cells and increased the proportion of cells in the G0 phase, thereby inhibiting proliferation. Co-culture with MSCs inhibited the proliferation and cell cycle progression of leukemia cells, while increasing the apoptosis rate, thus impairing the leukemogenic potential of AML cells both in vitro and in vivo. Mechanistic studies revealed that MMP14-mediated alterations in the AML stromal microenvironment are driven by PGE2 secretion and activation of the JAK-STAT pathway, promoting leukemia progression. Notably, inhibition of MMP14 can attenuate the chemotherapy resistance of AML cells induced by MSCs to cytarabine (Ara-C). Together, our study, for the first time, demonstrates the critical role of MSC-derived MMP14 in promoting AML progression and chemoresistance. Targeting MMP14 signaling pathways may offer novel therapeutic options for AML.

**Supplementary Information:**

The online version contains supplementary material available at 10.1186/s40164-025-00635-6.

## Background

The tumor microenvironment plays a crucial role in cancer progression and therapy resistance [1–3]. The bone marrow (BM) comprises a dynamic network of growth factors, cytokines, and stromal cells, fostering a conducive environment for leukemia development and progression [4]. Bone marrow-derived mesenchymal stem cells (BMSCs) are crucial components of the bone marrow microenvironment, capable of differentiating into osteoblasts, chondrocytes, and adipocytes under specific conditions [5, 6]. MSCs regulate both normal and malignant hematopoiesis, yet the underlying molecular mechanisms remain poorly understood. MSC function is markedly altered in AML patients. In addition to changes in growth kinetics and differentiation potential, MSCs from AML patients alter gene expression associated with the induction of hematopoietic progenitor cell quiescence [7, 8]. MSC-derived signaling within the bone marrow may elucidate the origin of bone marrow-derived changes observed in AML, which confer a leukemic advantage over normal hematopoiesis [9]. MSCs can regulate the development of leukemia through direct cell-to-cell contact, cytokine-receptor interactions, and exosome-mediated communication [10–13]. These interactions hinder physiological hematopoiesis while promoting LSC survival and proliferation. Therefore, further studies on MSCs within the bone marrow microenvironment are crucial for advancing therapeutic options for leukemia.

Matrix metalloproteinases (MMPs) are critical enzymes that degrade the extracellular matrix and disrupt tissue barriers, mediating changes in the tumor microenvironment during development and playing a crucial role in tumor invasion and metastasis [14–16]. As a transmembrane protein, MMP14 activates pro-MMP2 to induce tumor cell invasion. MMP14 is highly expressed in various tumors, promoting inflammation, angiogenesis, cancer cell invasion and metastasis, and drug resistance [17–21]. Additionally, aberrant expression of MMP family molecules has been studied in leukemia [22–24]. Despite the well-established significance of MMP14 in cancer, the regulatory mechanisms and functions of MSC-derived MMP14 in AML remain largely unexplored.

Here, we demonstrate the dynamic functional and molecular alterations of BMMSCs associated with AML burden. We also found that MMP14 is highly expressed in AML-MSCs, playing a crucial role in leukemia cell proliferation, apoptosis, and chemoresistance. Most importantly, depletion of MMP14 in MSCs leads to the inhibition of AML development. Mechanistically, MSC-derived MMP14 is a major contributor to the activation of the JAK-STAT pathway through PGE2 secretion, contributing to increased AML proliferation and chemoresistance. Aberrant expression of MMP14 in MSCs may aid in identifying novel therapeutic targets for AML treatment.

## Methods

### Drugs and reagents

The MMP14 inhibitor NSC 405020 was purchased from MCE (HY-15827, China). Cytarabine was purchased from Actavis (Italy). PGE2 was obtained from MCE (HY-101952, China).

### Cell culture

MSCs were derived from fresh BM aspirates of patients with newly diagnosed AML and healthy controls using a density gradient centrifugation with Ficoll-Lymphoprep™ (TBD, China). BMMSCs were maintained in Human MSC Growth Medium (Cyagen Biosciences Inc.) with regular medium changes every three days. After three days, non-adherent cells were removed through complete medium replacement, while the adherent cells were persistently cultured. All experiments utilized MSCs from passage 3. Surface immunophenotyping via flow cytometry characterized the MSCs. Morphological assessment confirmed the differentiation of MSCs into adipocytes and osteocyte lineages (Fig.S1). MSCs from C57BL/6 mice were acquired from Cyagen Biosciences Inc. These cells were propagated in Mouse MSC Growth Medium (Cyagen Biosciences Inc.) and utilized within five passages. AML cell lines Kasumi-1 and Molm-13, sourced from the Cell Resource Center of the Shanghai Institutes for Biological Sciences (Shanghai, China), were cultured in RPMI 1640 medium (HyClone, USA). All cells were incubated at 37 °C in a humidified atmosphere of 5% CO2.

### RNA library construction and sequencing

Total mRNAs were isolated from AML-MSCs (passage 3, *n* = 5) and HD-MSCs (passage 3, *n* = 5) using TRIzol reagent (Vazyme, China) and measured using NanoDrop (Thermo). RNA library construction and sequencing analysis were performed by Novogene (Tianjin, China). Genes with a p value < 0.05 were identified as differentially expressed genes (DEGs).

### Proteomic analysis of secreted proteins in BMSC culture supernatants

The MSC medium was replaced with FBS-free DMEM/F12 medium 24 h before supernatant extraction. Following 24 h of culture, the supernatant was harvested, spun down at 1500 rpm for 20 min to eliminate nonviable and dead cells, and preserved at -80 °C for further proteomic analysis. Samples derived from MSC supernatants underwent analysis on a Q Exactive mass spectrometer (Thermo Fisher Scientific, Bremen, Germany) by KangChen Bio-tech (Shanghai, China). MaxQuant software (version 1.5.6.0) was utilized to process raw data. Proteins exhibiting a differential expression p value < 0.05 were deemed significantly different.

### Immunofluorescence microscopy of BM sections

Bone marrow biopsy specimens were quickly placed in formalin fixative. Following a minimum of 4 h of fixation, the tissue underwent decalcification with EDTA for 24 h, proceeded by standard dehydration and paraffin embedding, producing sections of 5 μm thickness. Slides were treated with MMP14 (380861, Zenbio, China) and CD271 (55014-1-AP, Proteintech, China) antibodies for two days, then with 4’,6-diamidino-2-phenylindole (DAPI) for 12 h.

### Real-time quantitative RT-PCR (qRT-PCR)

Total RNA was isolated using Trizol (Vazyme, China) and converted into cDNA with Hifair^®^ II 1st Strand cDNA Synthesis SuperMix (Yeasen Biotechnology, 11120ES60). Quantitative RT-PCR analysis utilized the SYBR Green method via the CWBIO Fast SYBR Mixture real-time PCR system (CWBIO, China). qRT-PCR primer sequences can be found in Supplemental Table 1. Relevant gene expression levels were measured using the 2-ΔΔCt method.

### Lentivirus infection

MMP14 shRNA (shMMP14 group) and scrambled shRNA (control group) were obtained from Hanheng Biotechnology (Hanheng Biotechnology Co. Ltd., Shanghai, China). Green fluorescent protein (GFP) was used to assess transduction efficiency, and puromycin was used to select stably transduced cells.

### Cell proliferation, apoptosis and cell cycle analysis

Cell proliferation tests were carried out through flow cytometry to measure BrdU incorporation, executed as per the manufacturer’s instructions (BD Pharmingen BrdU Flow Kit, BrdU-APC). Apoptosis rates were determined by flow cytometry using the Apoptosis Detection Kit (Lianke Technology Co., Ltd.). Cell cycle analysis was performed using the Cell Cycle Analysis Kit (Lianke Technology Co., Ltd.).

### Colony forming Unit-Fibroblast (CFU-F) assay

The colony forming unit-fibroblast (CFU-F) assay was performed on two groups of MSCs by plating 2,000 cells from each group on 6 cm² dishes. The cells were cultured in MSC medium for 14 days with medium changes every three days. Following 14 days, the cells were preserved with 4% paraformaldehyde and dyed with crystal violet. The colony forming ability of MSCs was assessed by counting colonies containing more than 50 cells.

### Animal experimentation

MLL-AF9 retroviral transduction was conducted on CD117 + enriched cells from 8–12-week-old C57BL/6 mice, isolated via magnetic bead sorting (Miltenyi, 130-097-146, Germany), and subsequently infected with MSCV-MLL-AF9-IRES-YFP retroviruses twice amidst 8 µg/mL Polybrene (Hanheng Biotechnology Co. Ltd., HB-PB-500), 10 ng/mL IL-3 (PeproTech, 213 − 13), 10 ng/mL IL-6 (PeproTech, 216 − 16), and 20 ng/mL SCF (PeproTech, 250-03) in IMDM medium. Sub-lethally irradiated (7 Gy) recipient mice then received the infected cells via intravenous transplantation. Sorted by fluorescence-activated cell sorting (FACS), the MLL-AF9-expressing cells from primary recipient mice with AML were preserved for further transplantation to induce AML. A total of 100,000 MLL-AF9 cells in 200 µL PBS were intravenously transplanted into recipient mice. The MMP14 knockdown and control MSCs (100,000 cells/10 µL PBS) were then injected into the distal femora of both the right and left hind limbs. The mice were subsequently divided into two groups: the shMMP14 knockdown group and the control group. NSG mice (female, 5 weeks old) were procured from Jiangsu Jicui Biotechnology Co., Ltd. Primary human leukemia cells were isolated from high-leukemia AML patients using leukapheresis and PBMC separation. Each mouse was intravenously injected with 1 × 10^7 cells to induce leukemia. The successful establishment of the patient-derived xenograft (PDX) model was confirmed by observing leukemia cells in BM smears and through flow cytometry, with leukemia cell proportions in the BM ≥ 20%. Information on the antibodies used in the experiments is provided in Supplemental Table 2.

### Western blot

Proteins were isolated from cells using RIPA lysis buffer, which included PMSF and phosphatase inhibitors A and B. Protein aliquots were subjected to 10% SDS-polyacrylamide gel electrophoresis and then transferred onto PVDF membranes (Millipore Corp, Billerica, MA, USA). Overnight at 4 °C, the membranes were treated with primary antibodies (dilution 1:1000): MMP14 (380861, Zenbio, China), STAT5 (13179-1-AP, Proteintech, China), P-STAT5 (80115-1-RR, Proteintech, China), STAT3 (10253-2-AP, Proteintech, China), P-STAT3 (28945-1-AP, Proteintech, China), COX-2 (501253, Zenbio, China), and GAPDH (60004, Proteintech, China). Subsequently, for 1 h at room temperature, the membranes were treated with either goat anti-rabbit IgG (H + L) antibody (SA00001-2, Proteintech, China) or goat anti-mouse IgG (H + L) antibody (SA00004-1, Proteintech, China). An imaging system (Tanon, China) was used for detection.

### ELISA

Culture supernatants were collected from each well, and PGE2 protein levels were measured using the PGE2 ELISA kit (Jianglaibio, Shanghai, China). Duplicate wells were used for standard curves and experimental samples for each measurement. Concentrations were determined according to the manufacturer’s instructions.

### Histopathology

Femurs and tibiae from animal studies were utilized to prepare BM smears with Wright-Giemsa staining. Tissues obtained from mice underwent sectioning, dewaxing, and rehydration. Some samples received hematoxylin-eosin (HE) staining, while others underwent immunohistochemical analysis. Sections were treated with antibodies against P-STAT3 (CY6501, Abways) and P-STAT5 (CY5569, Abways). All slides were examined and photographed through a Nikon microscope outfitted with a high-resolution digital camera.

### Statistical analysis

Statistical analyses were performed using GraphPad Prism (8.0), gene set enrichment analysis (GSEA), and R scripts. Differences between groups were analyzed using two-tailed t-tests. All data are expressed as means ± standard deviations. Kaplan-Meier survival curves for mice were generated using the log-rank (Mantel-Cox) test in Prism 8.0. A p value of < 0.05 was considered statistically significant. *** *p* < 0.001, ** *p* < 0.01, and * *p* < 0.05.

## Results

### Multi-omic analysis identifies MMP14 as a key molecule in AMLMSC supporting leukemia cell growth

**Fig. 1:**
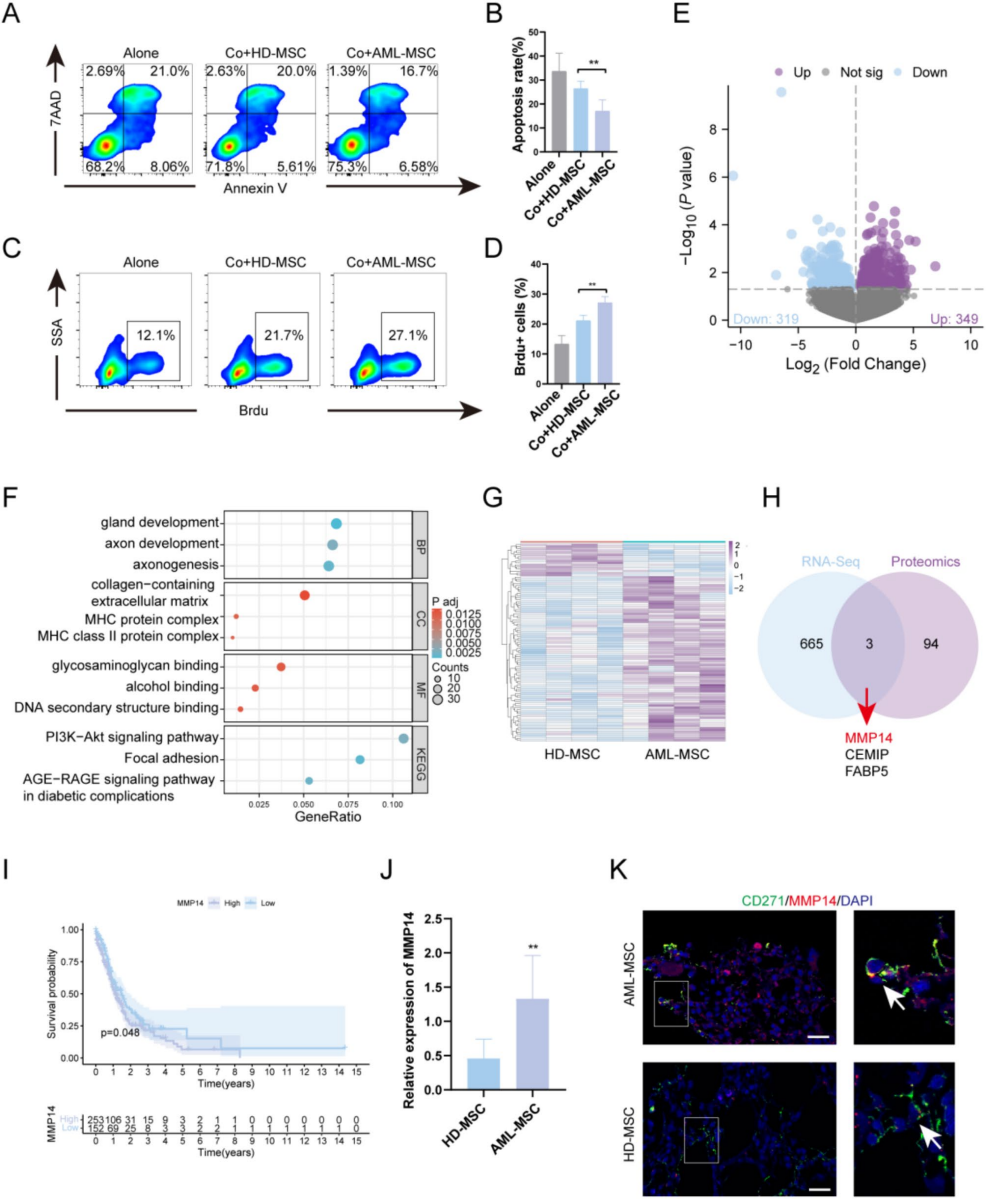
Combined transcriptomic and proteomic analyses identified MMP14 as a key molecule in AMLMSC supporting leukemia cell growth. **A**, **B**. Analysis of apoptosis in primary leukemia cells after 48 h of co-culture with five cases each of AML-MSC and HD-MSC, using the Annexin V APC Apoptosis Detection Kit (*n* = 5). Alone, leukemia cells cultured alone; Co, leukemia cells cocultured with MSCs. **C**, **D**. Flow BrdU assay for assessing the proliferation of primary leukemia cells after 48 h of co-culture with 5 AMLMSC and 5 HDMSC cases(*n* = 5). **E**. Volcano plots displaying the expression of differentially expressed genes between five AML-MSC and five HD-MSC samples, with purple representing upregulation and blue representing downregulation(*n* = 5). **F**. Enrichment analysis of differentially expressed genes. **G**. Proteomic analysis of supernatants from 4 AML-MSC and 4 HD-MSC samples, including a heat map of differentially expressed proteins(*n* = 4). **H**. Intersection analysis of differentially expressed genes identified in transcriptomic and proteomic analyses. **I**. Kaplan-Meier survival analysis of OHSU-AML dataset revealed that high expression of MMP14 is associated with poorer overall survival. **J**. PCR detection of MMP14 expression in AMLMSC (*n* = 12) compared to HDMSC (*n* = 6). **K**. Co-immunohistochemical staining of MMP14 (red) and CD271 (green) in AML and healthy control bone marrow samples. Scale bar = 20 μm (*n* = 5)

To investigate the capacity of AML-MSC and HD-MSC to support leukemia cell proliferation, primary leukemia cells were co-cultured with AML-MSC and HD-MSC for two days in vitro. Flow cytometry revealed a lower apoptosis rate in leukemia cells co-cultured with AML-MSC, while a Brdu assay indicated a higher proliferation rate in the AML-MSC group (Fig. 1A-D). Similarly, leukemia cell lines Kasumi-1 and Molm-13 were cultured with AML-MSC and HD-MSC for two days in vitro, which showed that AML-MSC, compared to HD-MSC, promoted leukemia cells proliferation and reduced apoptosis rates (Fig. S2). To further investigate the mechanisms by which AMLMSCs promote AML cell growth compared to HDMSCs, we collected BMMSCs from five patients with primary AML diagnosis and five healthy controls for transcriptome sequencing. Differentially expressed genes (DEGs) were identified between AML-MSCs and HD-MSCs with criteria of P value < 0.05. In total, 668 DEGs were detected, comprising 349 upregulated and 319 downregulated genes (Fig. 1E; Supplemental Table S3, 4). To further elucidate the pathways and processes influenced by the identified DEGs, GO and KEGG analyses were conducted. KEGG pathway enrichment analysis indicated significant enrichment in the PI3K-Akt signaling pathway and focal adhesion (Fig. 1F). Following the characterization of gene properties in AML-MSCs and HD-MSCs, we aimed to identify proteins characteristic of the AML-MSC supernatant. To identify secreted proteins, we conducted label-free proteomics on AML-MSCs and HD-MSCs. A total of 97 differentially expressed proteins were identified, comprising 81 upregulated and 16 downregulated proteins (P value < 0.05; Fig. 1G). Details of the altered protein expressions are provided in Supplemental Table S5. We integrated transcriptomic and proteomic analyses, revealing overlapping genes (Fig. 1H). To further screen candidate molecules, OHSU-AML survival analysis indicated that MMP14 was associated with poor prognosis in AML (Fig. 1I). We subsequently verified high MMP14 expression in AML-MSCs using RT-qPCR and immunofluorescence in bone marrow MSC specimens from AML patients compared to those from healthy donors (Fig. 1J, K). These data suggest that MMP14 is a key molecule in AML-MSCs supporting leukemia cell growth.

### Knockdown of MSC-derived MMP14 inhibits MSC proliferation and induces cytokine alterations

**Fig. 2:**
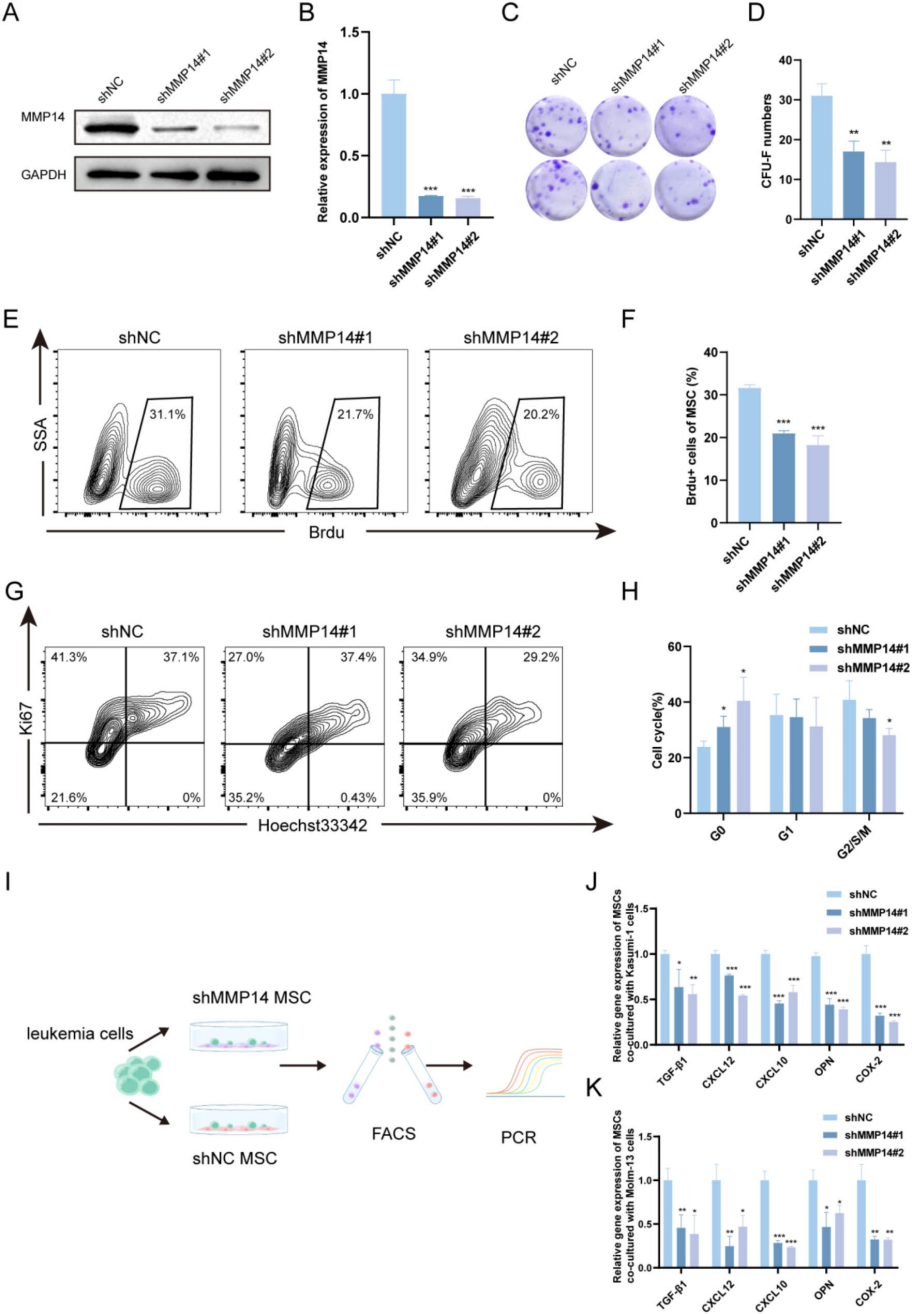
Effects of MMP14 on MSC proliferation and cytokine secretion. **A**, **B**. Knockdown efficiency of MMP14 in MSCs verified by Western blot and RT-qPCR. **C**, **D**. Representative plots of CFU-F in the MMP14 knockdown and control groups. Quantification of CFU-F colonies in MSCs. **E**, **F**. BrdU assay showing cell proliferation rates in the MMP14 knockdown and control groups. **G**, **H**. Cell cycle analysis of the MMP14 knockdown and control groups. **I**-**K**. After co-culturing Kasumi-1 and Molm-13 cells with MSCs from the knockdown and control groups for 48 h, flow sorting was performed on the MSCs followed by PCR analysis of TGF-β1, CXCL12, CXCL10, OPN, and COX-2 gene expression levels

To evaluate the impact of MMP14 upregulation in AMLMSC on MSCs, shRNA constructs (shMMP14#1/#2) were employed to inhibit MMP14 expression. Both shRNAs significantly reduced MMP14 expression at the protein and RNA levels (Fig. 2A, B). CFU-F assay revealed that MMP14 knockdown decreased the colony-forming ability of MSCs (Fig. 2C, D). A subsequent BrdU assay demonstrated reduced proliferation of MSCs in the MMP14 knockdown group (Fig. 2E, F). Cell cycle analysis via flow cytometry was conducted to determine if MMP14 knockdown-induced inhibition was attributable to cell cycle disruption. The analysis indicated a significant increase in the G0 phase of MSCs (Fig. 2G, H). Studies have demonstrated that MSCs can modulate the bone marrow microenvironment through cytokine secretion, which is closely related to AML pathogenesis and progression [25, 26]. Therefore, we investigated whether MMP14 knockdown alters cytokine secretion by MSCs. After co-culturing MSCs from the knockdown and control groups with Kasumi-1 cells and Molm-13 cells for 48 h, we performed flow cytometric sorting of MSCs and PCR detection of leukemia progression-related genes (Fig. 2I and Fig. S3). The results showed significant reductions in TGF-β1, CXCL12, CXCL10, OPN, and COX-2, of which COX-2 and OPN have been shown to be associated with promoting leukemia progression [10, 27], and CXCL12, TGF-β1, and CXCL10 have been reported to be associated with leukemia resistance [13, 28, 29](Fig. 2J, K).

### Knockdown of MSC-derived MMP14 significantly inhibits AML progression in vitro

**Fig. 3:**
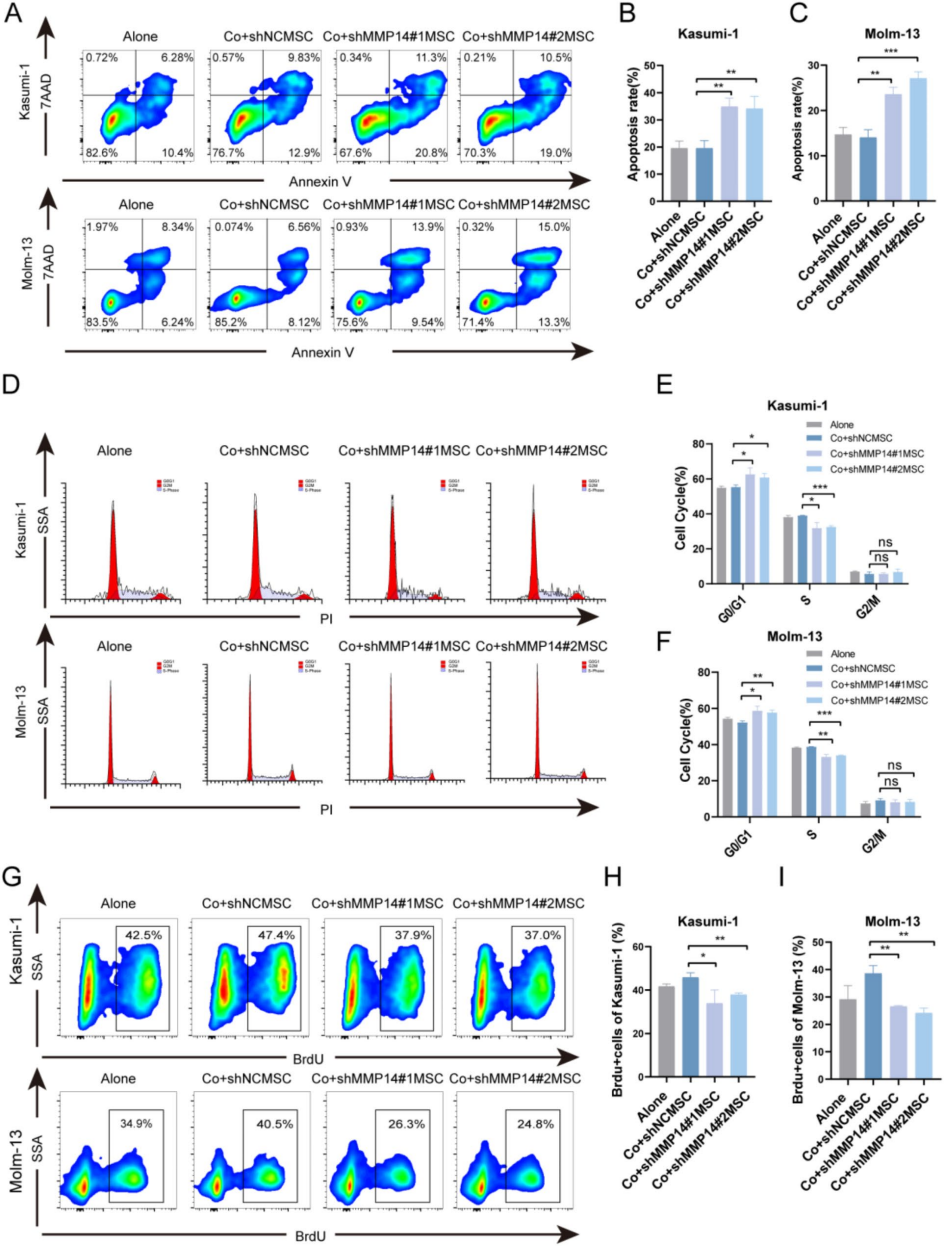
Knockdown of MSC-derived MMP14 significantly inhibits AML progression in vitro. **A**-**C**. Flow cytometry analysis of apoptosis rates in Kasumi-1 and Molm-13 cells co-cultured with the knockdown and control groups for 48 h. **D**-**F**. Flow cytometry PI analysis of cell cycle changes in Kasumi-1 and Molm-13 cells co-cultured with the knockdown and control groups for 48 h. **G**-**I**. Flow cytometry BrdU analysis of cell proliferation rates in Kasumi-1 and Molm-13 cells co-cultured with the knockdown and control groups for 48 h

To elucidate the effect of MSC-derived MMP14 on AML progression, we co-cultured two AML cell lines, Kasumi-1 and Molm-13, with MSCs transduced with either sh-MMP14 or sh-NC. We observed several changes in the AML cells, notably an increase apoptosis rate in both Kasumi-1 and Molm-13 upon MMP14 knockdown (Fig. 3A-C). Additionally, cell cycle analysis revealed that MMP14 inhibition in MSCs increased the proportion of cells in the G0/G1 phase while decreasing the proportion in the S phase (Fig. 3D-F). Furthermore, a BrdU assay demonstrated that MMP14 inhibition led to reduced proliferative capacity in both Kasumi-1 and Molm-13 cells (Fig. 3G-I). These findings suggest a potential role of MSC-derived MMP14 in the pathogenesis of AML.

### Inhibition of MSC-derived MMP14 delays AML progression

**Fig. 4:**
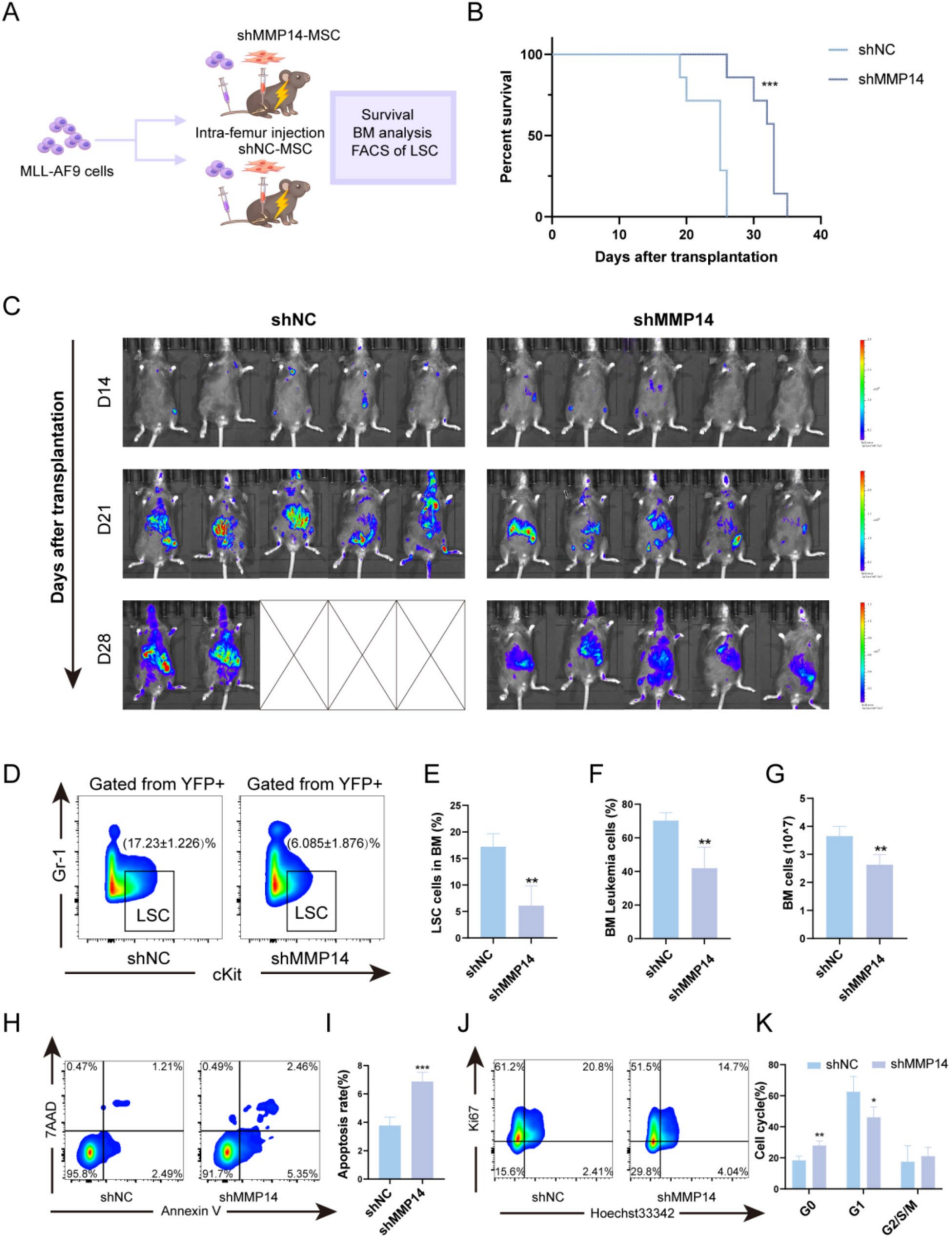
Effect of MMP14 deletion in MSCs on disease progression and leukemic stem cells in MLL-AF9 leukemic mice. (**A**) The experimental layout involved transplanting MLL-AF9 cells via the tail vein and MSCs from MMP14 knockdown and control groups via intra-femoral injection into mice. AML progression was monitored by flow cytometric analysis. (**B**) Survival rates of mice in MMP14 knockdown and control groups after cell transplantation (*n* = 7). (**C**) Live imaging was performed on days 14, 21, and 28 post-transplant to monitor disease progression and burden(*n* = 5). **D**, **E**. Flow cytometry plots of BM LSCs and statistical analysis of the proportion of LSC in whole BM (*n* = 4). **F**. Statistical analysis of the proportion of leukemia cells in mice of both groups (*n* = 4). **G**. Bone marrow cell count statistics in both groups of mice (*n* = 4). **H**, **I**. Representative flow cytometry plots of LSC apoptosis and statistical analysis of the apoptosis rate of LSCs (*n* = 4). **J**, **K**. Representative flow cytometry plots of the LSC cell cycle and statistical analysis of the percentage of apoptosis in LSCs (*n* = 4)

We further established an animal model of AML to investigate the role of MSC-derived MMP14 in AML disease progression. Bone marrow YFP + cells were isolated from virus-transduced leukemia mice with MLL-AF9/YFP. These MLL-AF9 cells were transplanted into 7 Gy-irradiated mice via the tail vein and injected intrafemurally with BMMSCs from two groups: lentivirally-transfected MMP14 knockdown and control mice (Fig. 4A). We first examined whether MMP14 deletion in MSCs affected the survival of MLL-AF9 AML mice. The results, as shown in Fig. 4B, indicated that leukemia progression was delayed and survival was significantly prolonged following MMP14 knockdown. Additionally, tumor progression was monitored weekly using bioluminescent imaging, revealing that the tumor burden in the MMP14 knockdown group was lower than that in the control group(Fig. 4C). Further comparison of the infiltration of leukemic cells (YFP + cells) and leukemic stem cells (LSC YFP + c-Kit + Gr-1-) in the two groups of mice revealed that the proportion of bone marrow leukemic cells and LSCs in the MMP14 knockout group was lower than in that the control group (Fig. 4D-G). Additionally, LSC characterization showed increased proportions of apoptotic cells and G0 phase cells in the MMP14 knockdown group (Fig. 4H-K). These results suggest that inhibition of MSC-derived MMP14 can effectively delay AML progression.

### MMP14 exerts its functions by activating the JAK-STAT pathway in AML cells through the secretion of PGE2

**Fig. 5:**
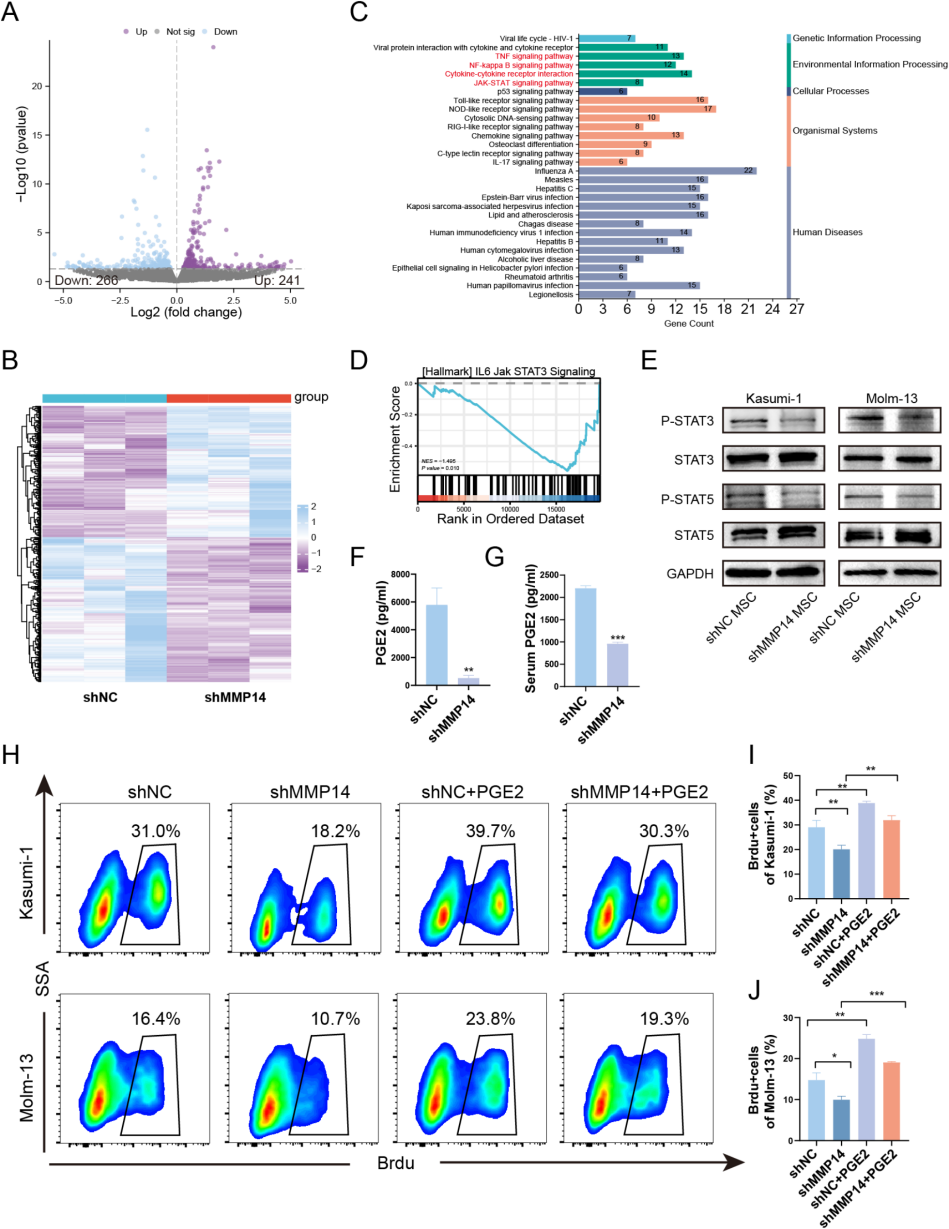
MMP14 exerts its functions by activating the JAK-STAT pathway in AML cells through the secretion of PGE2. (**A**) Transcriptome analysis of Kasumi-1 cells co-cultured with MSCs from the MMP14 knockdown and control groups after 48 h. Volcano plots are shown. (**B**) Heat map of differentially expressed genes. (**C**) Enrichment analysis of differentially expressed genes. (**D**) GSEA analysis showing that MMP14 knockdown in MSCs is associated with inhibition of the JAK-STAT pathway. (**E**) Western blot analysis of STAT3, P-STAT3, STAT5, and P-STAT5 protein levels in Kasumi-1 and Molm-13 cells co-cultured with MSCs from the MMP14 knockdown and control groups. (**F**) ELISA assay of PGE2 levels in the supernatant of MSCs from the MMP14 knockdown and control groups. (**G**) ELISA assay of PGE2 levels in the serum of mice from the MMP14 knockdown group and the control group. H-J. Proliferation of Kasumi-1 and Molm-13 cells in the presence or absence of PGE2 (1 μm) in the co-culture system of the knockdown and control groups

To elucidate the potential mechanisms of MSC-derived MMP14 in AML development, we conducted RNA-seq analysis on Kasumi-1 cells co-cultured with MMP14-knockdown and control MSCs, with each group comprising three biological replicates. The volcano plot and heat map indicate that 507 genes were differentially altered, with 241 genes upregulated and 266 genes downregulated (Fig. 5A, B). KEGG enrichment analysis revealed that MMP14 knockdown in MSCs was associated with the TNF signaling pathway, NF-kappa B signaling pathway, cytokine-cytokine receptor interaction, and JAK-STAT signaling pathway (Fig. 5C). GSEA analysis indicated that MMP14 knockdown inhibited the JAK-STAT signaling pathway in Kasumi-1 cells (Fig. 5D). Therefore, we analyzed the expression of STAT3, P-STAT3, STAT5, and P-STAT5 in Kasumi-1 and Molm-13 cells co-cultured with MMP14-knockdown and control MSCs using Western blotting. As shown in Fig. 5E, knockdown of MMP14 in MSCs resulted in a decrease in the expression of P-STAT3 and P-STAT5 in leukemia cells, with no decrease in the expression of total STAT3 and STAT5. Given that studies have shown that MMP14 can regulate COX-2 expression, our data verified this as well. Western blotting showed that MMP14 knockdown in MSCs resulted in decreased expression of COX-2, a key enzyme in PGE2 production (Fig. S4). Additionally, the literature reports that downregulation of PGE2 is associated with inhibition of the JAK-STAT pathway [30]. Therefore, we hypothesized that MMP14 knockdown resulted in decreased PGE2 secretion by MSCs, which in turn inhibited the JAK-STAT pathway in AML cells. To this end, we examined PGE2 levels in the supernatants of MSCs from both groups. ELISA results showed that MMP14 knockdown in MSCs led to reduced PGE2 levels. Additionally, we assessed the PGE2 levels in the serum of mice from the MMP14 knockdown and control groups, finding that PGE2 levels were decreased in the serum of mice with MMP14 knockdown (Fig. 5F-G). To validate whether MSC-derived MMP14 exerts its effects through the secretion of PGE2, we added PGE2 to the co-culture system and observed that PGE2 could reverse the inhibition of cell proliferation caused by MMP14 knockdown (Fig. 5H-J).To further delineate the regulatory role of PGE2 on the JAK/STAT pathway, the expression of P-STAT3 and P-STAT5 was assessed using flow cytometry in the presence or absence of PGE2 within the co-culture systems of the knockdown and control groups. The results demonstrated that the knockdown of MMP14 in MSCs significantly inhibited the JAK/STAT pathway, an effect that was reversed by the addition of PGE2(Fig. S5). These data suggest that MSC-derived MMP14 promotes AML progression by activating the JAK-STAT pathway in AML cells through PGE2 secretion.

### MMP14 confers chemoresistance to Ara-C

**Fig. 6:**
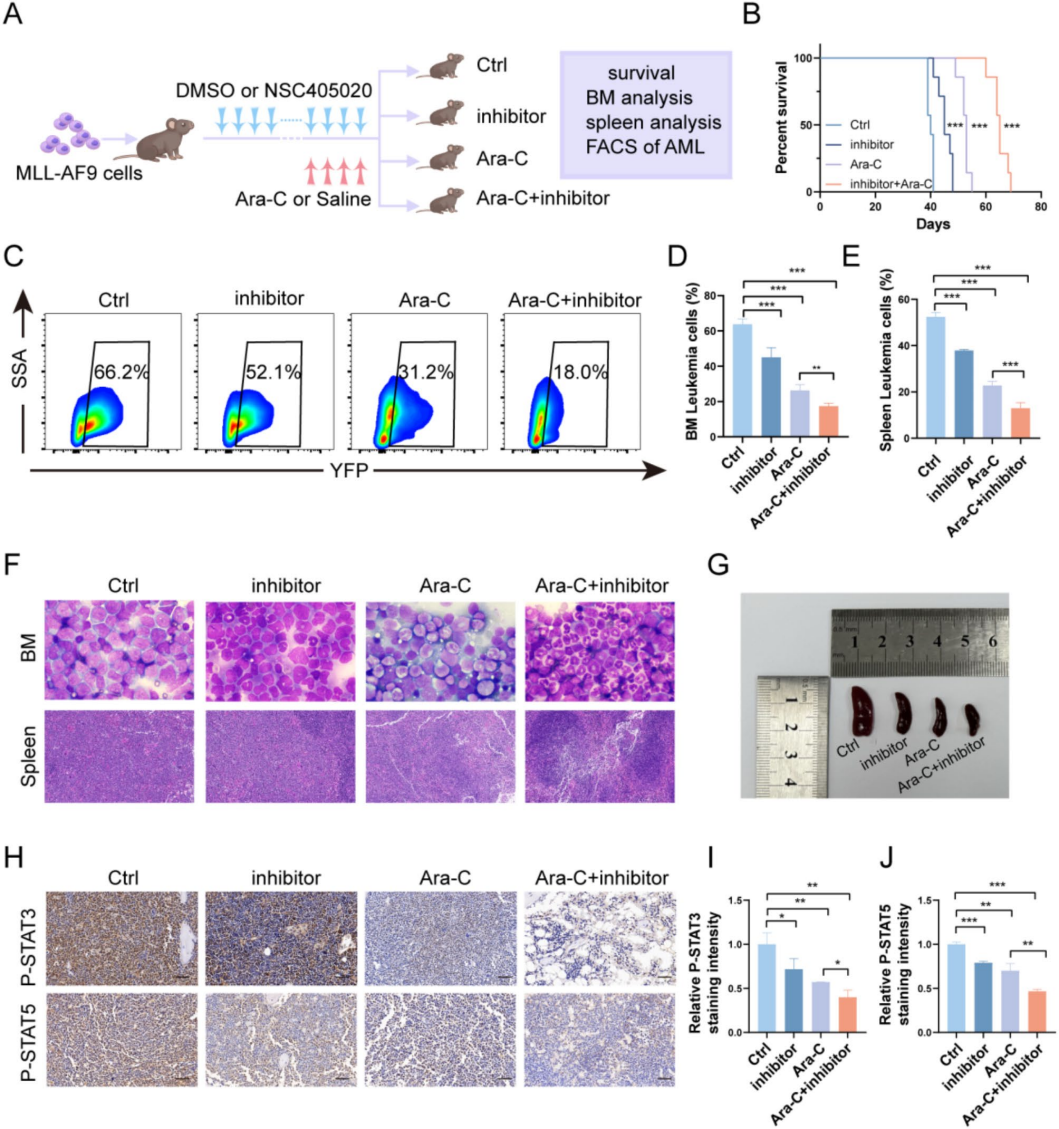
MMP14 confers chemoresistance to Ara-C. **A**. Overview of the experimental design, dividing mice into four groups based on the presence or absence of the inhibitor (NSC-405020) and cytarabine. **B**. Survival statistics of mice (*n* = 7). **C**-**E**. Representative FACS analysis and statistical proportions of leukemia cells in the four groups of mice (*n* = 4). **F**. Representative images of Wright-Giemsa staining of bone marrow cells and hematoxylin-eosin staining of spleens. **G**. Representative images of mouse spleens. **H**-**J**. Immunohistochemical staining of P-STAT3 and P-STAT5 in mouse bone marrow and quantification of the staining. Scale bar, 50 μm

Mesenchymal stem cells play a crucial role in protecting leukemia cells from chemotherapeutic agents. Studies have shown that activation of the JAK/STAT pathway may be involved in the mechanism of cytarabine resistance in AML [31]. Therefore, we investigated the effect of MMP14 deletion on chemosensitivity to cytarabine. To this end, we established the MLL-AF9 leukemia model by intrafemoral injection of MMP14 knockdown or control MSCs, followed by tail vein injection of MLL-AF9 cells. The mice were subsequently divided into four experimental groups, with or without Ara-C treatment: the shNC group, the shMMP14 group, the shNC combined with Ara-C group, and the shMMP14 combined with Ara-C group (Fig. S6A). Survival analysis revealed that survival time was significantly prolonged in the shMMP14 group, the shNC combined with Ara-C group, and the shMMP14 combined with Ara-C group, with the latter exhibiting the most pronounced survival benefit (Fig. S6B). Bone marrow smears and flow cytometric analysis further demonstrated that leukemic burden was more effectively suppressed in the shMMP14 group treated with Ara-C, compared to the shNC control group (Fig. S6C-F). These findings suggest that the inhibition of MSC-derived MMP14 enhances the cytotoxic effects of Ara-C in vivo. To explore the clinical relevance, we co-cultured Kasumi-1 and Molm-13 leukemia cell lines with MSCs in vitro, and treated the co-cultures with either the specific MMP14 inhibitor NSC-405,020 [32]or Ara-C to evaluate the combinatorial effect. Flow cytometric analysis of apoptosis revealed that both monotherapies and combination treatments induced apoptosis in a concentration-dependent manner, with the combination of the MMP14 inhibitor and Ara-C exhibiting a synergistic effect (CI < 1) (Fig. S7A-D). Additionally, Western blotting analysis of key proteins in the JAK/STAT pathway demonstrated that the combination therapy significantly reduced the levels of P-STAT3 and P-STAT5, thereby inhibiting the activation of the JAK/STAT signaling pathway(Fig. S7E).Furthermore, to investigate the therapeutic potential in vivo, we administered MMP14-specific pharmacological inhibitors (NSC-405020) to mice, dividing them into four groups: DMSO-treated control (Ctrl), MMP14 inhibitor (inhibitor), cytarabine (Ara-C), and MMP14 inhibitor combined with cytarabine (inhibitor + Ara-C). The experimental design is illustrated in Fig. 6A. At the conclusion of the experiment, the highest survival rate was observed in the MMP14 inhibitor combined with cytarabine group (Fig. 6B). Additionally, the proportion of bone marrow and splenic leukemia cells in mice was assessed by flow cytometry. The proportion of bone marrow and splenic leukemia cells in mice treated with the combination of MMP14 inhibitor and cytarabine was significantly lower than in the control and single-agent groups (Fig. 6C-E). Furthermore, bone marrow smears demonstrated a significant reduction in leukemia cells in the MMP14 inhibitor combined with cytarabine group, and infiltration of splenic leukemia cells was diminished as evidenced by histological H&E staining (Fig. 6F). Spleen size was significantly reduced in the MMP14 inhibitor combined with cytarabine group (Fig. 6G). Reductions in P-STAT3 and P-STAT5 were more pronounced in the combination therapy group compared to the monotherapy group (Fig. 6H-J). These data suggest that MMP14 confers resistance to Ara-C chemotherapy.

### Inhibition of MMP14 suppressed AML patient blasts

**Fig. 7:**
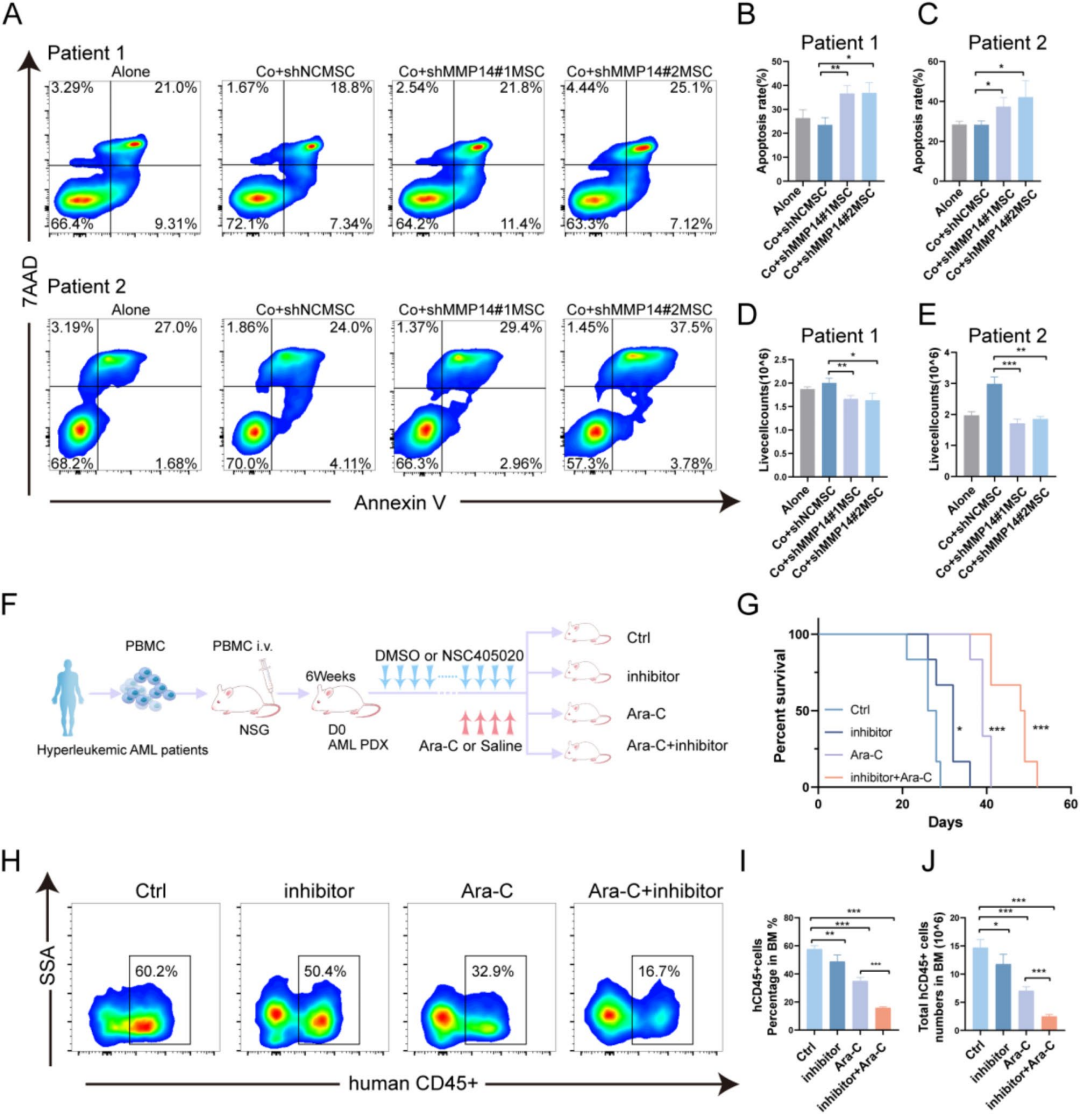
Inhibition of MMP14 suppressed AML patient blasts. **A**-**C**. Flow cytometry analysis of apoptosis rates in primary leukemia cells co-cultured with the knockdown and control groups for 48 h. Alone, leukemia cells cultured alone; Co, leukemia cells cocultured with MSCs. **D**, **E**. Viable cells were counted using trypan blue staining and assessed with a hemocytometer after 48 h of co-culturing. **F**. Schematic of the PDX model construction process. Primary human leukemia cells were isolated from high-leukemia AML patients using leukapheresis and PBMC separation. These cells were then injected into NSG mice via the tail vein. Four weeks later, one mouse per week was randomly sacrificed, and the proportion of leukemia cells in the BM was analyzed by flow cytometry. Drug treatment commenced when the leukemia cell proportion reached ≥ 20%. Following successful PDX model construction, mice were randomly allocated to four treatment groups: DMSO-treated control (Ctrl), MMP14 inhibitor (inhibitor), cytarabine (Ara-C), and a combination of MMP14 inhibitor and cytarabine (inhibitor + Ara-C). **G**. Survival statistics for the mice were compiled (*n* = 6). H-J. Flow cytometric representative plots and statistical analysis detailing the proportion and absolute numbers of human CD45 + cells in the four mouse groups (*n* = 5)

To further elucidate the role of MSC-derived MMP14 in primary leukemia cells, we obtained cells from two distinct patients and co-cultured them with MMP14 knockdown and control MSCs. Flow cytometry analysis of apoptosis revealed that knockdown of MMP14 increased the apoptosis rate in primary leukemia cells (Fig. 7A-C). Additionally, we employed trypan blue staining to count the viable cells after co-culture, and the results showed that the viable cell counts of primary cells co-cultured with MSCs from the knockdown group were lower than those of the control group (Fig. 7D, E). We subsequently assessed the impact of MSC-derived MMP14 knockdown on normal bone marrow-derived CD34 + cells and showed that knockdown of MMP14 did not significantly affect apoptosis and proliferation of normal CD34 + cells (Fig. S8). To evaluate the in vivo effect of MMP14 knockdown on AML blasts, patient-derived cells were transplanted into NSG mice to establish a PDX model. This model was stratified into four groups: DMSO-treated control (Ctrl), MMP14 inhibitor (inhibitor), cytarabine (Ara-C), and MMP14 inhibitor combined with cytarabine (inhibitor + Ara-C) (Fig. 7F). The results revealed that in vivo inhibition of MMP14 expression prolonged the survival of PDX mice. More importantly, the combined MMP14 inhibitor and cytarabine group had a significant survival advantage compared to the inhibitor or cytarabine alone (Fig. 7G), and the results showed that the inhibitor of MMP14 group reduced the leukemia burden by flow cytometric analysis of human CD45 + cells in the BM of PDX mice, the combination of the administered drug group had the lowest proportions and absolute numbers of human CD45 + cells, consistent with our findings in the mouse model of MLL-AF9 (Fig. 7H-J). In conclusion, our findings suggest that inhibition of MMP14 suppresses AML patient blasts, underscoring its potential as a therapeutic target in leukemia.

## Discussion

**Fig. 8:**
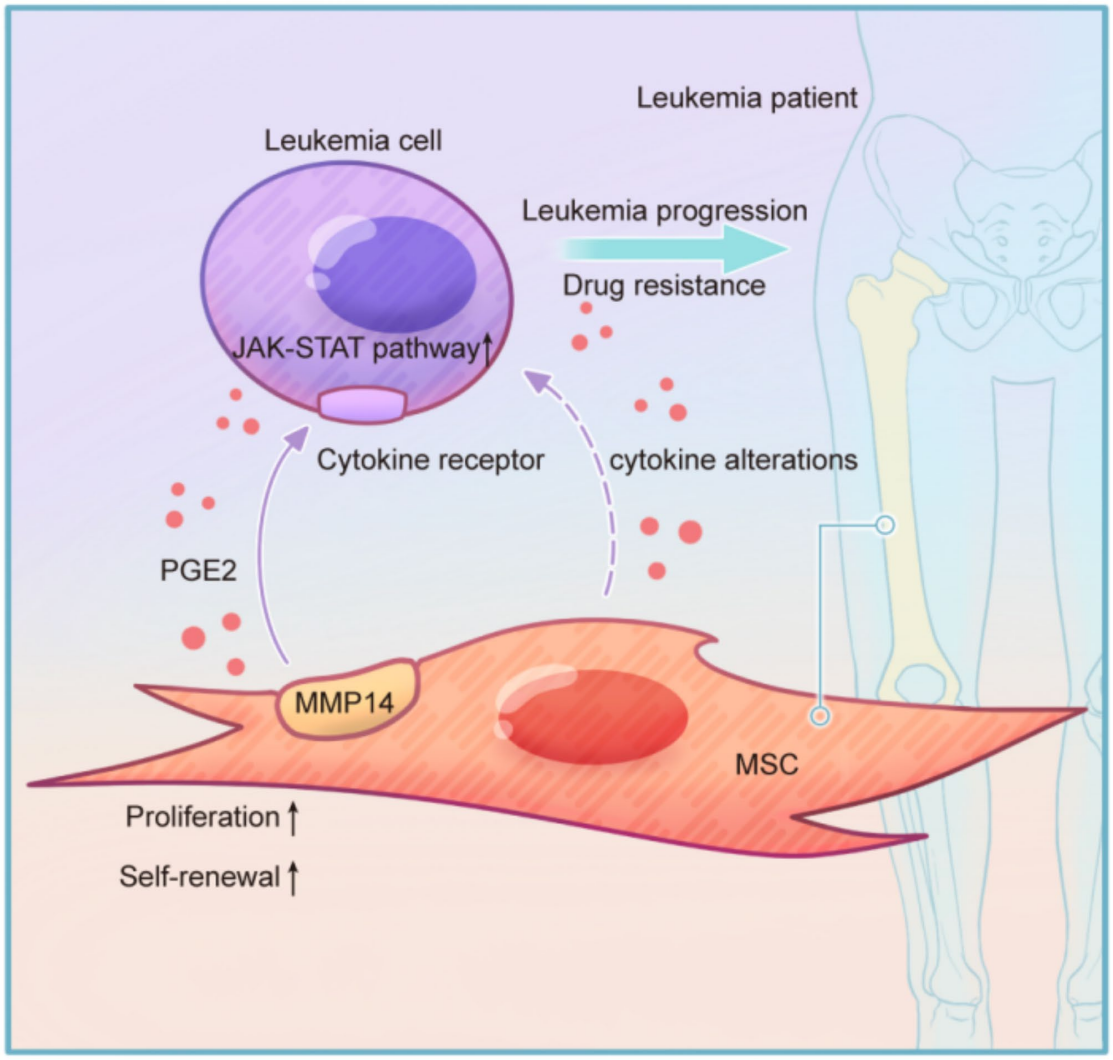
Mechanism of action of MSC-derived MMP14 in promoting AML progression. The model illustrates how MSC-derived MMP14 promotes AML cell growth and chemotherapy resistance through the secretion of PGE2, activating the JAK-STAT pathway, leading to AML progression. Additionally, MSC-derived MMP14 enhances MSC proliferation and self-renewal, while also inducing cytokine alterations

The contribution of the bone marrow microenvironment to leukemogenesis and progression has been increasingly recognized [33–36]. However, the specific contribution of BM MSCs to leukemia niche formation and progression remains unclear. Therefore, understanding the role of BM MSCs in leukemia is crucial for identifying disease-specific therapeutic targets. MMP14 is elevated in numerous cancers and facilitates tumor angiogenesis, inflammation, and progression [24, 37]. Although there is substantial evidence that MMP14 is aberrantly expressed in leukemia [22, 38], the role of MSC-derived MMP14 in AML and its impact on the microenvironment remain unclear. Here, we demonstrate that AML-MSCs are molecularly and functionally distinct from normal donor-derived MSCs. For the first time, we demonstrate that MSC-derived MMP14 promotes MSC proliferation and accelerates AML progression. Furthermore, in vivo experiments demonstrated that inhibiting MMP14 increased chemosensitivity to cytarabine in AML mice. Mechanistically, the supportive effect of MSC-derived MMP14 on AML may operate through the secretion of PGE2, which activates the JAK-STAT pathway in leukemia cells (Fig. 8). This underscores the pivotal role of MSC-derived MMP14 in AML pathogenesis and progression, offering fresh evidence for the involvement of MSCs in AML development.

In this study, we co-cultured AML-MSCs and HD-MSCs with leukemia cells in vitro to compare their ability to support AML cells, and found that AML-MSCs were more supportive of AML cell growth compared to HD-MSCs. Previous studies have reported differences among the MSCs from leukemia patients compared to those from healthy donors [39–42]. Additionally, numerous studies have shown that interactions between leukemia cells and cellular components in the bone marrow microenvironment are key in chemoresistance, clonal proliferation, and cell growth, as well as in disease relapse [10, 43]. The contribution of underlying molecular alterations in AML-MSCs to leukemogenesis and chemoresistance remains controversial, emphasizing the need to study these cells further. To elucidate why AML-MSCs support leukemia cell growth, we performed transcriptomic and proteomic analyses, along with joint OHSU-AML datasets prognostic analysis, which preliminarily confirmed that MMP14 plays a key role in AML development and progression.

Accumulating evidence strongly suggests that MSCs play a critical role in leukemia progression, primarily through the secretion of specific cytokines, growth factors, and chemokines. Carter et al. revealed that co-culturing AML cells with MSCs significantly increased COX-2 expression and PGE2 production in MSCs, thereby promoting leukemia cell growth via the β-catenin signaling pathway [10]. Moreover, Xiao et al. identified that OPN is significantly upregulated in the AML bone marrow microenvironment, contributing to the formation of a leukemic niche that supports AML cell survival and proliferation [27]. Additionally, the CXCL12-CXCR4 axis plays a pivotal role in AML progression and drug resistance, with studies indicating that disruption of this axis can enhance the efficacy of targeted therapies [44, 45]. Research indicates that TGF-β1 secreted by MSCs enhances the resistance of AML cells to cytarabine therapy through the TGF-β/p38/ALDH2 signaling pathway [28]. Furthermore, CXCL10 was shown to promote leukemia cell survival and drug resistance by enabling cells to evade apoptosis induced by chemotherapeutic agents like cytarabine and methotrexate [29]. Building upon these insights, we selected several molecules closely associated with leukemia progression and examined the impact of MMP14 knockdown on the gene expression of cytokines in MSCs. Our results are consistent with published studies, though further investigation is warranted to elucidate the functional implications. The role of PGE2 as an autocrine and paracrine signaling molecule has been well documented in cancer, where it promotes tumor cell proliferation, survival, invasion, and migration [46]. The JAK/STAT signaling pathway, a downstream pathway of cytokine signaling, is involved in crucial biological processes such as cell proliferation, differentiation, apoptosis, and immune regulation. It is strongly linked to the progression of hematological malignancies [47, 48].Our data show that MSC-derived MMP14 activates the JAK-STAT pathway in leukemia cells by regulating PGE2 secretion.

Drug resistance remains a central challenge in treating AML. Despite recent advances in targeted therapies and the development of drugs for specific gene mutations, the efficacy of current treatments continues to face significant hurdles [49, 50]. Emerging research suggests that interactions between LSCs and associated stromal cells critically influence leukemia onset, progression, and therapy response. Within the bone marrow niche, stromal cells provide a sanctuary that allows LSCs to develop drug-resistant phenotypes and evade chemotherapy-induced death. Cytarabine serves as a first-line agent in AML treatment. While cytarabine-based regimens can achieve complete remission in the majority of newly diagnosed AML patients, overall clinical outcomes are often unsatisfactory due to persistent drug resistance. Multiple mechanisms are strongly associated with resistance to cytarabine [51–53]. For instance, targeting the bone marrow microenvironment, specifically Lama4, has been shown to mitigate Ara-C chemoresistance in AML cells [35]. Our study reveals that MSC-derived MMP14 bestows cytarabine resistance in AML LSCs. Mechanistically, inhibiting MMP14 in AML models enhances sensitivity to cytarabine by disrupting the JAK-STAT pathway.

Dissecting the crosstalk between leukemia cells and the BM microenvironment will not only enhance our understanding of leukemogenesis but also help identify new strategies to target microenvironment-mediated LSC survival and drug resistance. Future studies should explore the effects of MSC-derived MMP14 on bone marrow microenvironment components and hematopoiesis. In conclusion, our study provides the first evidence of the effect of MSC-derived MMP14 on AML progression. Additionally, MSC-secreted MMP14 promotes MSC proliferation and self-renewal, while also inducing cytokine alterations. MSC-derived MMP14 may promote AML progression and increase drug resistance through the secretion of PGE2, which activates the JAK-STAT pathway in leukemia cells. Therefore, strategies to inhibit MSC-derived MMP14 may represent a promising therapeutic approach to inhibit AML and sensitize AML LSCs to chemotherapy, thereby improving treatment outcomes.

## Conclusions

Our study provides the first evidence that MSC-derived MMP14 promotes AML cell growth and chemotherapy resistance via the secretion of PGE2, which activates the JAK-STAT pathway, consequently leading to AML progression. Additionally, MSC-derived MMP14 enhances MSC proliferation and self-renewal, concurrently inducing alterations in cytokine profiles. Targeting the MMP14 signaling pathway may offer new therapeutic options for AML.

## Electronic supplementary material

Below is the link to the electronic supplementary material.

Supplementary Material 1

## Data Availability

The datasets used and/or analysed during the current study are available from the corresponding author on reasonable request.

## Abbreviations

LSCs: leukemic stem cells
MSCs: mesenchymal stromal cells
AML: acute myeloid leukemia
AML-MSCs: AML patient-derived MSCs
HD-MSCs: healthy donor-derived MSCs
GFP: Green fluorescent protein
Ara-C: cytarabine
BMSCs: Bone marrow-derived mesenchymal stem cells
CFU-F: Colony Forming Unit-Fibroblast
DEGs: Differentially expressed genes
FACS: fluorescence-activated cell sorting
PDX: patient-derived xenograft
