# Supplementary material for: MMP14 from BM-MSCs facilitates progression and Ara-C resistance in acute myeloid leukemia via the JAK/STAT pathway
Source: Exp Hematol Oncol. 2025 Mar 22;14:43. doi: 10.1186/s40164-025-00635-6 (PMC11929205; doi:10.1186/s40164-025-00635-6)
Supplement: Supplementary file 1 — Supplementary Material 1 [file 40164_2025_635_MOESM1_ESM.pdf]

Supplementary Tables

Table S1 Primers used in quantitative real-time PCR assay

Gene	Forward primer (5'-3')	Reverse primer (5'-3')
MMP14	CCTTGGACTGTCAGGAATGAGG	TTCTCCGTGTCCATCCACTGGT
TGF-β1	GGCCAGATCCTGTCCAAGC	GTGGGTTTCCACCATTAGCAC
CXCL12	CTCAACACTCCAAACTGTGCCC	CTCCAGGTACTCCTGAATCCAC
CXCL10	TGAAAAAAGAAGGGTGAGAAGAGATG	CCTTTCCTTGCTAACTGCTTTCAG
OPN	CGAGGTGATAGTGTGGTTTATGG	GCACCATTCAACTCCTCGCTTTC
COX-2	CTTCCTCCTGTGCCTGATGATTG	CCCTCGCTTATGATCTGTCTTGA

Table S2 Technical specifications of the antibodies (clones and sources) employed in flow cytometry analysis

Antibody	Conjugate	Clone	Source	Catalog No
anti-human CD105	PerCP/Cyanine5.5	43A3	Biolegend	323215
anti-human CD19	PE	HIB19	Biolegend	302207
anti-human CD34	FITC	581	Biolegend	343503
anti-human CD45	FITC	HI30	Biolegend	982316
anti-human CD11b	FITC	ICRF44	Biolegend	982614
anti-mouse Ly-6G (Gr-1)	PE	RB6-8C5	Biolegend	108407
anti-mouse CD117 (c-kit)	PE/Cyanine7	ACK2	Biolegend	135111
anti-mouse Ki-67	APC	16A8	Biolegend	652405
anti-human CD45	PE	HI30	Biolegend	304008

TableS3. General information of AML patients and healthy controls.

Patient ID	Diagnostic	Cytogenetics	Molecular	Age (years)	Gender
AML01	AML-M5	46, XY	-	30	M
AML02	AML-M2	46, XY	CEBPA， NPM1， FLT3ITD	38	M
AML03	AML-M2	46, XX	MLL-PTD	23	F
AML04	AML-M5	46, XY	NPM1exon12	65	M
AML05	AML-M2	46, XX t (11;19) (q23; p13.3)	MLL-ELL，NRAS G12D，FLT3ITD	16	F
HD01	normal	46, XX	-	48	F
HD02	normal	46, XX	-	23	F
HD03	normal	46, XY	-	39	M
HD04	normal	46, XX	-	52	F
HD05	normal	46, XY	-	66	M

HD, healthy donor; M, male; F, female; –, no mutations found for FLT3, NPM1, CEBPA, and IDH1.

Table S4 Differential expression genes between AML-MSC and HD-MSC.

gene_name	log2FoldChange	pvalue
GSTM5	-6.46828	2.73E-10
GSTM1	-10.6609	8.81E-07
IMPA2	1.576737	1.66E-05
GLP2R	3.414528	2.77E-05
MTX1P1	1.284632	4.95E-05
CFI	-3.31965	6.02E-05
MSLN	2.375387	8.54E-05
COL4A4	3.33868	8.94E-05
SFRP4	-2.21951	0.000127
AGRN	1.764627	0.000171
SSTR1	-2.86624	0.000186
SMN1	-1.34037	0.000201
PCBP3	2.375922	0.000207
FBXO32	1.324689	0.000212
ITGA3	1.043759	0.000239

HLA-DRA	-5.57847	0.000247
NGFR	3.493056	0.000269
BCAM	0.974079	0.000271
CELSR1	2.2515	0.000378
AL589743.4	4.6546	0.00044
CCL5	5.196149	0.000497
TRHDE	3.71023	0.000626
TRPC4	-1.77277	0.000695
NES	-2.05795	0.000709
EGR1	-2.1361	0.000796
AP001542.3	2.269102	0.000807
DDX58	2.66209	0.000812
AL355032.1	-2.9837	0.000845
AEBP1	-1.60623	0.000865
TNFSF9	1.652184	0.001015
RARRES1	-2.16805	0.001018
BEND5	-2.28952	0.001048
LINC01060	-1.87441	0.001075
SUSD3	-2.49589	0.001086
CADM3	3.432157	0.001182
TGFB2-AS1	2.038273	0.001225
ADAMTSL5	1.113966	0.001232
CD74	-4.2025	0.001255
TRPC6	-2.24165	0.001293
TGFB2	1.790728	0.001307
HLA-DMB	-3.91595	0.001403
PCDHGA3	1.657502	0.001442
COL4A3	2.97552	0.001484
AC002401.4	2.356279	0.001535
ZNF826P	-1.4979	0.001601
WFDC21P	1.890798	0.001677
AC020763.3	1.88765	0.00169
LXN	-1.46518	0.001718
ADARB1	1.263485	0.001739
ADAP1	2.169573	0.001864
STRA6	-3.57569	0.001892
SCN2A	-2.89122	0.001918
LBP	-4.36345	0.002
KCNJ12	1.619345	0.002231
HCK	3.674927	0.002262
PTK2B	1.158417	0.002411
NCF2	1.73035	0.002577
RPL22L1	-1.53741	0.002599
BATF2	3.890676	0.002602

ARHGEF28	1.446233	0.002658
PENK	-2.23204	0.002692
HLA-G	-2.97572	0.002754
LAMA3	-1.98641	0.002795
PRKCE	0.973708	0.002813
PEG13	3.521486	0.002833
CGNL1	1.745808	0.002858
AC147651.3	-4.29317	0.002869
CLDN1	2.004377	0.00294
ZNF618	0.779347	0.002999
HGF	-2.01084	0.003016
MMP14	0.788737	0.003154
HLA-DPA1	-3.35667	0.003209
LCNL1	2.915213	0.003228
BPI	-4.10075	0.003448
CDCA7	-2.95157	0.00346
KIAA1324L	2.488826	0.003533
PCDHGB7	1.007171	0.003623
EFHD1	2.072753	0.003632
HLA-L	-1.71846	0.003779
ASB5	1.850539	0.003834
HLA-DMA	-2.23492	0.003972
ITPR1	1.303019	0.003995
DYSF	1.524407	0.004063
KRTAP1-1	-2.44852	0.004176
PGM5	3.096271	0.004216
NUTM2G	2.552275	0.004262
DDX60L	2.007817	0.004386
RGS7BP	-2.81606	0.004456
AC114489.1	4.155929	0.004574
PCDHGB2	1.196462	0.004625
NEO1	0.754418	0.004675
RHOU	-1.86745	0.004746
LEP	3.661878	0.004753
MCM10	-2.61956	0.0048
LINC00862	1.741888	0.004843
ZHX2	0.719799	0.004849
IGFBPL1	1.870157	0.005251
SMILR	-1.1614	0.005281
KRT31	-3.75532	0.005293
FRG1JP	-3.58546	0.005331
TSHZ2	-2.46417	0.005364
KLHL24	0.890386	0.005366
TCP11L2	0.892213	0.005373

AC078817.1	-3.37513	0.005418
PLD5	6.917401	0.00547
CHRNE	-1.68909	0.005601
GASAL1	1.193508	0.005603
RIMS3	1.930034	0.00562
ARMC9	0.723916	0.005717
FMNL1	1.75533	0.005723
AC087163.2	2.956039	0.005966
HAS2	-1.27192	0.006072
CCKAR	-2.89483	0.006171
KCNQ5	-1.73843	0.006179
HMGA2	-1.02083	0.006234
FBLN7	-0.90705	0.006286
POLR2J3	-1.44538	0.006294
CYGB	-1.42125	0.00639
AC007878.1	3.198122	0.006395
BMF	1.362988	0.006418
RND2	-2.04106	0.006455
OXCT2P1	-1.84144	0.006574
GREM2	-1.26961	0.006592
AC018804.1	-1.56712	0.006592
CPXM2	-1.96632	0.006676
PCDHGA2	1.097317	0.006797
FAM216A	-0.68389	0.006883
POU3F3	2.266007	0.006925
SCFD2	-0.86637	0.007043
HAAO	-2.85307	0.007215
LAMA5	1.511737	0.007265
MPP7	1.798972	0.007279
SLPI	2.496253	0.007359
AC138230.1	-3.7733	0.00736
ANKFN1	1.646644	0.007375
DNER	4.09601	0.007414
AL161431.1	4.784212	0.007655
CARD11	3.784911	0.007691
ARHGAP26	0.961492	0.008003
ABCG2	-2.32671	0.008106
PIK3AP1	3.480345	0.008175
SLC6A6	1.435844	0.008215
ACER2	1.441657	0.008257
CHRM4	3.941193	0.008293
NTNG1	-2.71838	0.008306
CSPG4P13	3.605596	0.008332
SPRY4-AS1	-1.88619	0.008363

FOXN3	1.018463	0.00847
IFFO2	1.015595	0.00855
BCL2L11	1.451546	0.008625
TRPC3	-3.01662	0.008666
SUPT16HP1	-3.35928	0.008721
PDK4	2.999842	0.009296
AC008946.1	-1.78613	0.009356
FAM162B	-4.54476	0.009366
LBH	1.097788	0.009453
EXO1	-2.32699	0.009467
TRIM16L	0.768575	0.009728
TFB1M	-0.98814	0.009747
FP565260.3	3.637729	0.009898
S100B	-2.68564	0.009945
AC246817.1	2.971276	0.009972
TMEM97	-1.53685	0.01004
ARHGAP23	0.747413	0.010042
NID2	1.003079	0.01017
OLFM2	-1.67882	0.010175
MMP16	-1.63152	0.010279
AL020996.1	1.443829	0.010387
SLFN13	-1.64133	0.010418
PKIB	1.371306	0.01044
ALPL	-1.82986	0.010443
AP002387.1	-1.98737	0.010527
UBE2H	0.574746	0.010654
FGD6	1.020318	0.010659
FOXL2	1.934976	0.011076
FAM86GP	-3.22808	0.011116
PLN	-3.15867	0.011309
AC026358.1	-3.95612	0.011483
MST1P2	1.713122	0.011599
AC020898.1	-3.09251	0.01162
SHC3	2.320817	0.011629
CD200	-1.26992	0.011734
HSPB8	1.06297	0.011874
DTL	-1.98818	0.011899
CASP7	0.824199	0.011917
PPP2R2C	1.875697	0.011958
AKAP12	-1.04106	0.012022
ITGA11	1.035478	0.012071
BAIAP2L2	-1.13648	0.012116
AC004556.1	1.633739	0.012166
FPR1	1.313917	0.012218

HECW2	-1.49136	0.012397
MTSS1L	0.580683	0.012399
NLRP3	2.170718	0.012541
PTPRM	0.726045	0.012691
IL33	-6.92627	0.012759
ST3GAL1	0.724745	0.012931
BEX1	-1.91769	0.012945
ZIC4	-2.59861	0.012991
BMS1P10	-2.61731	0.013016
DOK5	-1.0459	0.01327
HMGN1P38	-1.24681	0.013286
ABHD4	0.800305	0.013524
TSPAN7	-2.34176	0.013729
FO393414.3	-2.69248	0.013828
NAA80	-0.79397	0.013934
EBP	-1.37396	0.013956
RPS3AP5	-2.05083	0.013998
IQCA1	1.578266	0.014073
DCLK1	-1.25359	0.014091
GPR157	0.82601	0.014132
PROB1	1.0174	0.014143
AL590004.3	1.586459	0.01421
NAGS	-1.24961	0.014225
C2orf16	0.709055	0.014275
DAPK1	0.913838	0.014682
COL4A1	1.132237	0.01471
EXT1	-0.65972	0.014741
PLCXD1	-0.72392	0.014802
FANCA	-1.53128	0.014847
NRCAM	1.360549	0.01487
CCL28	-0.99795	0.014899
APBB1IP	0.682936	0.014904
LINC02407	1.522395	0.015072
NKX2-2	4.254782	0.015114
RPS26P3	-1.79344	0.015274
AFMID	-0.71618	0.015281
COL4A5	2.122868	0.015287
PLXND1	0.843191	0.015296
CDC14B	0.536705	0.015357
GPR62	3.722751	0.015386
APOL6	1.043803	0.015513
COMMD3	-0.91318	0.015689
SMARCD3	-0.54916	0.015711
AC127496.7	1.093491	0.0159

ADGRE2	-1.42251	0.015956
SNHG15	-0.63463	0.016006
MANF	-0.78763	0.016119
SERPINH1P1	3.761148	0.016135
PTGER2	-1.59012	0.016433
FABP3	-1.69295	0.016562
BRIX1	-1.60788	0.01658
AL645939.5	-1.70744	0.01672
E2F2	-2.44428	0.016766
LIF	-1.44252	0.016975
STON2	2.628037	0.017043
AC006058.3	2.18889	0.017086
PLSCR4	0.859662	0.01713
C9orf170	2.09542	0.017174
PPM1L	1.403648	0.017295
FZD4	0.892467	0.017344
DDR1	0.634086	0.017376
AC004585.1	1.395781	0.017381
ALDOC	-1.22891	0.017385
LINC01134	1.582725	0.017424
POLE2	-1.42214	0.017464
NCOA3	1.08084	0.017478
DCLK2	1.271745	0.017591
PYGM	-1.56927	0.017681
HKDC1	1.357969	0.017739
THEMIS2	1.949266	0.01793
SERPINI1	1.133744	0.018034
MAPK13	-0.6922	0.018145
AC023043.1	-1.87316	0.018214
ADAMTSL1	-0.93646	0.018388
ACAT2	-1.96664	0.018561
PARP14	1.555289	0.018635
ABLIM2	1.932594	0.01864
AL445648.1	3.859752	0.018674
OTOGL	1.452287	0.018793
SDR42E1	1.342481	0.018916
PRKXP1	2.160592	0.019101
AL591845.1	-1.73476	0.019184
PCNA	-0.7414	0.019335
GFOD1	1.021908	0.019471
PURG	2.294491	0.01949
AC004837.2	3.60693	0.01952
CCDC74B	-1.09389	0.019523
LINC02223	2.055926	0.019531

ZNF208	-2.06359	0.019579
EMP1	-0.70771	0.01969
CMSS1	-0.50976	0.019716
FLT4	4.232083	0.019751
PKMYT1	-1.73452	0.019764
FHAD1	2.019587	0.019799
SLC5A3	1.599597	0.019813
LIN7B	-0.93796	0.019926
LRFN4	0.69739	0.019928
MOXD1	-1.61118	0.019929
AC010343.1	-1.79816	0.020009
RAD51D	-0.60879	0.020107
KYAT3	-0.82336	0.020277
CEMIP	1.283079	0.020358
NAPRT	-1.23294	0.020503
PLCD3	0.711567	0.020518
TRABD2A	-1.03688	0.020636
KCNC3	1.212686	0.020864
MFAP3L	1.356113	0.02094
ATP1A2	2.378757	0.021098
MALL	-3.35577	0.021114
SEL1L3	0.719838	0.021126
ERG28	-1.53039	0.021147
HELLS	-1.80006	0.021153
TM4SF20	2.799435	0.021245
IRX2	0.97845	0.021252
POLA2	-0.81289	0.021401
LYPD1	-1.25687	0.021454
MYL3	-2.91376	0.021478
ZSWIM5	-1.57131	0.021615
PDE4A	0.52146	0.021646
MDK	-1.5335	0.02165
SNORA65	-3.09647	0.021691
ISL2	1.457915	0.021705
ADARB2	2.988548	0.021834
ETV1	-1.37262	0.021855
WWP2	0.637903	0.021881
STARD9	1.164029	0.021988
PCAT7	2.362218	0.022047
NOG	1.896454	0.022205
RAET1E	1.918733	0.02223
PALD1	-1.38072	0.022266
AC073046.1	2.488676	0.022267
CRELD2	-0.75414	0.022632

AC135977.1	-2.42755	0.022637
AC113383.1	0.967864	0.023051
KRT9	-2.04055	0.023055
CLSPN	-2.12453	0.023144
CYP51A1P2	1.498533	0.023232
MYH14	2.596413	0.023316
AC025569.1	1.13391	0.023318
PPIAP31	-1.44885	0.023326
PADI1	1.672811	0.023375
KISS1	3.174732	0.023397
AC254633.1	-1.65138	0.023416
AC106782.1	-2.09441	0.023461
NUDT8	-1.14849	0.023499
PARVB	-0.58584	0.023583
IGF2R	0.878665	0.023599
ALDH1A3	-1.33048	0.023811
KRT23	-2.0368	0.023814
TRPM8	3.82952	0.023838
PFN1P1	-1.11846	0.023942
CELSR2	0.739278	0.024115
AC139149.1	1.386692	0.024307
GDF7	3.110245	0.024615
DCHS2	1.768286	0.024625
KRT14	-1.75805	0.0247
SAA1	3.114202	0.02476
UPF3AP1	2.712008	0.024778
SLC26A11	0.425967	0.024824
CCDC58	-0.66097	0.024885
METTL7B	-2.06217	0.024925
CDC6	-1.15935	0.02501
SLC22A23	0.960361	0.025081
TAS1R1	1.902444	0.025213
GAPDHP65	-3.29368	0.025443
CELF5	2.171002	0.025484
COL4A2	0.917191	0.025541
PRL	-2.61919	0.025579
KRTAP1-5	-1.53035	0.025614
AC078881.1	-2.74073	0.025692
RPL12P4	-1.40388	0.026081
CHODL	-2.23655	0.026157
NDUFA4L2	-1.54524	0.026238
EVL	-0.72478	0.026252
CLIP4	0.731171	0.026442
ESPN	2.590827	0.026562

C3orf80	-2.392	0.026642
ABCG1	2.170743	0.026707
MSMO1	-1.54923	0.026715
KIF12	-3.80382	0.026827
PAAF1	-0.74727	0.026895
CFDP1	-0.48247	0.02691
ANKRD29	-1.08194	0.02691
AC023509.4	2.398538	0.027107
RBP4	-2.43089	0.027184
PINLYP	-1.74028	0.027197
C2CD4D	2.633422	0.027269
C9	2.361114	0.027292
AC005703.6	2.833722	0.027295
C8orf58	-0.89135	0.027687
GINS2	-1.26319	0.027897
AC079298.3	1.726069	0.02793
MBNL3	-1.17735	0.028073
XRCC2	-1.41522	0.028088
TM7SF2	-1.81407	0.028091
CTSC	-0.95022	0.028163
CDK5RAP2	0.616202	0.028185
RCAN3	0.91392	0.028208
KLHDC7B	1.647057	0.028224
PXDN	0.692416	0.028367
RASA4DP	-2.02741	0.028407
IL21-AS1	-3.82604	0.028428
TM6SF1	2.024352	0.028443
KCNK2	-0.84944	0.028539
WDR18	-0.72745	0.028549
AL031665.2	-2.92581	0.028612
AP003071.4	1.12357	0.028641
RSPO1	1.672196	0.028675
UHRF1	-1.25569	0.028717
KIAA1522	0.529574	0.02885
RNF152	1.032011	0.028862
RAP1GAP	-1.86415	0.028887
HCFC1R1	-1.32385	0.028925
AC245100.3	1.839356	0.028942
PCDHGB5	0.942624	0.028978
ASL	-0.62624	0.029026
HK3	3.891194	0.029159
COX7A1	-1.28454	0.029167
MMP23B	-2.09964	0.029281
GOLGA8G	3.341647	0.029308

AL136452.1	1.890521	0.02943
TTC28-AS1	0.87274	0.029451
LINC00702	-0.95142	0.029609
DLGAP1-AS2	1.175573	0.029698
MHENCR	-0.9063	0.029755
LINC01423	-1.83993	0.029851
YWHAEP1	-1.3533	0.030004
ARL4C	1.145919	0.030114
AL162258.2	-3.65563	0.030157
PRSS16	3.199223	0.030252
XDH	1.578847	0.030352
ATAD5	-1.49154	0.030417
GRIP2	2.048375	0.030439
TMEM25	-0.77732	0.030558
MYOM1	1.134366	0.03085
AK5	-1.30372	0.030975
RARA	0.489333	0.031146
SUMO2P3	-3.78735	0.031393
ESRP2	-3.17867	0.031431
MLPH	-1.31043	0.031432
DSCC1	-0.77188	0.031462
APOE	-2.4236	0.031595
KCNC4	-0.87317	0.031842
SEMA7A	1.287279	0.031852
ATP6V0A4	3.36736	0.031913
NKAIN2	1.387762	0.031949
TGM5	2.889339	0.032086
GUCY1A2	2.148144	0.032092
KCNE4	0.735595	0.03214
RPL37AP1	-1.82588	0.032234
C11orf91	-2.49954	0.032259
STAT3	0.514178	0.032312
CD37	1.167518	0.032324
DDIAS	-1.77556	0.032428
GSTT2	-1.86228	0.032605
AL080250.1	-1.80032	0.03261
PRR3	-0.67065	0.032627
AP001266.2	3.189692	0.032651
PCDHGA9	1.151134	0.032674
AL445250.1	-2.36434	0.032884
SPAG4	-1.08309	0.032889
ADRA1B	1.158406	0.032893
CLEC14A	-1.89392	0.032922
PCDHGA5	0.95215	0.033008

GNS	0.37177	0.033071
ABCA1	1.202848	0.033089
KIF5C	2.029985	0.033213
LINC01114	1.305961	0.033234
AL513497.1	1.435925	0.033264
NPTX1	2.06642	0.033305
UNC5B	0.957644	0.033315
AC011043.1	-1.03048	0.033428
FAM57A	-0.6816	0.033563
PRXL2A	-2.04746	0.033625
AC138951.1	4.418127	0.033678
HNRNPD	-0.53824	0.033727
C2orf88	-1.32218	0.033778
CCL7	2.018091	0.033997
DPF1	-1.08659	0.034088
DNAH6	2.397628	0.034187
RASSF5	1.131778	0.034406
PDGFC	-0.6945	0.034496
PSPHP1	-1.65475	0.034566
WEE2-AS1	1.087268	0.034627
TNFSF10	2.936683	0.034646
PF4	-3.06414	0.03465
VGF	2.256377	0.034814
PDGFRB	0.715717	0.034911
CH17-340M24.3	-1.24511	0.034981
MAP3K1	0.909789	0.035062
AC004461.2	2.902785	0.035101
CTRC	3.725136	0.035178
AP001160.2	-3.16203	0.035254
CHAF1B	-1.08146	0.035364
ZNF541	2.023422	0.035406
BTG3	-0.60386	0.035419
POLQ	-1.95253	0.035697
AC069148.1	2.252186	0.035709
TENM2	1.498421	0.035789
FAM111B	-2.8207	0.03618
AC011944.2	3.420108	0.036212
MCAM	1.326181	0.036264
SNRPA	-0.84441	0.036447
LCA5L	1.196471	0.036475
LINC01703	-1.17303	0.036566
CDC45	-1.81137	0.036589
SUSD2	-1.84138	0.036598
AMIGO2	1.604576	0.036673

SEMA3F	1.120338	0.03671
CDT1	-1.05236	0.036736
ARHGEF18	0.71823	0.036794
GABBR2	1.802015	0.036817
BCL10	-0.46535	0.03684
NLRC5	0.85241	0.036875
DMRTA1	1.535274	0.036913
PCSK6	1.173278	0.036956
CYP19A1	1.823428	0.037047
MITF	0.765939	0.037095
B4GALT5	0.823248	0.037168
SH3D21	-1.59727	0.037191
AP001107.5	-1.31576	0.037193
TUBA1B	-0.84321	0.037359
PI16	-2.49571	0.037362
EIF3C	0.85754	0.037422
EME1	-1.4864	0.037504
DPY19L2P2	-1.51909	0.037558
WDYHV1	-0.73953	0.037596
E2F8	-2.10906	0.037601
RPL24P4	-1.21207	0.037619
ADAR	0.757334	0.037641
PCDHB14	0.782568	0.037772
AADAC	-3.28038	0.03786
KCNN3	2.972488	0.037871
AGMAT	-1.04865	0.038049
COL6A6	2.735959	0.038163
PRRG1	0.765604	0.038204
SMIM10L2B	-1.3984	0.038251
DPY19L1P1	0.883332	0.038335
LDLRAD4	1.525713	0.038404
LMNTD2	1.008066	0.038434
SLC4A3	0.666414	0.038444
SIK1B	1.623521	0.03848
ANOS1	-0.91027	0.039002
SRM	-0.65606	0.039095
RNU6-722P	-1.76666	0.03914
DHRS3	-0.85168	0.039151
PPP2R2B	-3.73266	0.039264
AC092535.4	2.868213	0.039339
EEF1A1P19	-1.17282	0.039448
GYG2P1	-3.33319	0.039466
PARP9	1.150957	0.03948
NANOS1	1.131789	0.039562

PGF	-1.15305	0.039672
SMAP2	-0.60722	0.039687
CSF1	0.872549	0.039708
SORBS1	1.458742	0.039783
TRHDE-AS1	2.76534	0.039955
LINC00460	2.075256	0.040065
EPHB2	1.272149	0.040108
SNX29	0.447945	0.04012
HAUS1	-0.53031	0.040226
CDKN2B	0.806581	0.040246
B3GNT10	0.911875	0.040287
ACSS2	-1.24129	0.040298
ZNF367	-1.74407	0.040408
SP110	1.117961	0.040483
SERF1B	1.671458	0.040522
RPL41P2	-2.0623	0.040685
ZNRF2	0.549282	0.040739
IL7R	1.382641	0.040904
LATS2	0.633518	0.041212
ABCA3	0.87781	0.041238
RNF208	-0.73142	0.041266
ZBTB32	2.315739	0.041375
RAD51C	-0.53904	0.041397
INHBB	1.496337	0.041489
SAXO1	-1.45078	0.041568
LUARIS	1.828644	0.041674
AC098829.1	3.118502	0.041695
KIF15	-1.38056	0.041867
LINC00612	3.283152	0.041874
SPTBN4	-0.61805	0.041917
AC004264.1	-1.78807	0.041927
SULT4A1	1.295567	0.042224
ACTN2	4.285266	0.042226
RPS6KA1	0.80691	0.042247
ANTXR1	0.73002	0.042279
LINC02021	-1.92365	0.042423
LTB	2.88694	0.042616
FAM149A	-0.8618	0.042664
AC097065.1	2.615538	0.042874
ABCA4	1.415187	0.042903
ANGPT4	1.556349	0.042917
NUS1P1	1.613375	0.042943
GALNT18	-1.08389	0.04306
AKR1B10	1.703059	0.043107

SLC7A4	1.942056	0.043135
FAM69B	-0.96011	0.04317
USP43	2.952056	0.043186
AC013468.1	2.969347	0.043293
EEF2KMT	-0.57176	0.043476
IGSF3	1.81973	0.04352
C17orf53	-0.95695	0.043665
GTF2H2	-0.99576	0.043911
RPL15P2	-1.74079	0.043946
SCG5	-1.18847	0.044059
SPRY1	-1.30727	0.04406
MYO15A	-1.14037	0.044253
AC100788.2	3.496675	0.044434
AC069155.1	3.015927	0.044546
RXFP1	1.77267	0.044576
BLM	-1.70595	0.044595
FADS1	-1.03812	0.044672
ARHGAP5	1.090864	0.044773
MCM6	-0.86664	0.044774
AC084117.1	-2.03298	0.044815
PCDHGA11	1.148013	0.044894
LINC00452	1.416119	0.044906
NACA3P	-3.21414	0.044966
ABCB9	-0.64627	0.04507
FABP5	-0.9863	0.045086
AC120036.3	1.675585	0.045146
GALE	-0.79662	0.045435
PPP1R26-AS1	1.047652	0.045593
RASEF	4.067332	0.045621
SREBF2-AS1	-1.03201	0.045658
RPS10P3	-1.69678	0.045803
ZHX1-C8orf76	-2.26547	0.045887
AC096888.1	1.840833	0.0459
AC023794.2	-2.33094	0.045952
TLCD2	0.664677	0.046169
ZNFX1	0.846988	0.04657
GDF5OS	-3.01081	0.046584
WDR76	-1.28118	0.046637
HSD17B7	-1.38624	0.046737
HPRT1	-0.55741	0.046782
CAV1	-0.6523	0.046808
AL445183.2	3.152823	0.046857
AC044860.1	1.483444	0.046896
AL162727.1	3.220465	0.046898

AP004782.1	1.949493	0.047032
PTH1R	-1.32338	0.047037
TMEM217	1.146502	0.047115
HHEX	1.146616	0.047289
PTPRO	1.777063	0.047547
SQSTM1	0.70317	0.047645
STK38	0.49686	0.047737
FIBIN	0.991193	0.047773
NDUFA9	-0.69559	0.047892
NOS3	-2.24134	0.047906
LINC00908	-3.01685	0.04791
PIK3CD	0.592348	0.047951
PANTR1	2.544375	0.047984
PCDHB6	-1.28914	0.048011
CHST15	0.727956	0.048071
QPRT	-2.49114	0.048169
C5AR2	0.934953	0.048174
TUBB	-0.65228	0.048217
AL137060.1	1.320508	0.048239
AC011450.1	1.428157	0.04843
SAAL1	-0.47457	0.048931
HOTAIR	-0.8263	0.049049
TMEM107	-0.70244	0.049186
SLC16A14	1.411416	0.049187
AFAP1L1	1.098772	0.049226
PLTP	-0.76123	0.049241
AC246787.1	-1.34941	0.049437
RPTN	-3.06332	0.04944
RAB3IP	0.755412	0.049501
LMO7DN-IT1	2.24882	0.049606
FN3KRP	-0.40418	0.049764
AC124067.2	-2.41612	0.04978
ADRA1D	-1.33308	0.049788
CHKA	0.715865	0.049797
FDPS	-1.15156	0.049839
CAMKMT	-0.72215	0.049855
CETN2	-0.68077	0.049893

Table S5 Differential expression proteins between AML-MSC and HD-MSC.

id	logFC	P.Value
TSPYL2	3.455938	2.69E-05
PTMS	3.841957	5.91E-05
DLD	-3.72023	8.58E-05
FABP5	3.536213	9.00E-05
PSMA3	3.195492	0.000184
ARPC2	3.494086	0.000238
UCHL1	3.412253	0.000311
PROCR	-3.02637	0.000399
NBL1	4.283142	0.00042
NEDD8	2.957094	0.000674
CCT6B	-6.78103	0.000903
RBM15	-4.07631	0.000958
CRIM1	2.724534	0.001039
TPT1	3.558937	0.001127
NME1	3.107015	0.001221
LGALS3	3.032755	0.001237
WDR1	3.605937	0.001309
ACTR2	3.01602	0.001404
HAPLN3	3.708559	0.001438
PGLS	2.868285	0.001762
METRNL	2.401627	0.002124
UGP2	2.977439	0.002306
GSN	3.317438	0.003114
PVR	2.748884	0.003134
ITGB1	2.000679	0.004147
LUM	-1.95483	0.006019
VEGFA	2.951018	0.006413
SPEF2	-5.34092	0.006659
PLA2G2E	3.720002	0.006843
LASP1	2.396865	0.007441
ATOX1	1.89095	0.008079
PRCP	-1.6324	0.008597
RNASET2	1.987994	0.008765
PIP	-1.77585	0.008877
MANBA	4.635255	0.009814
RAD23B	1.730825	0.010292
DSTN	2.396646	0.011357
LTA4H	1.550829	0.012034
CORO1C	3.599282	0.012549
MMP14	1.993512	0.01299
TSN	2.13234	0.013144
PRNP	2.000478	0.013564

LY96	1.700438	0.01372
ANXA1	-1.81468	0.013742
IFI30	2.240432	0.013756
NACA	1.671664	0.016717
PGM1	2.101123	0.017119
UBE2N	1.55885	0.017942
HMCN1	3.838057	0.01855
ADH1C	3.70056	0.019487
RAP1B	-2.07658	0.02005
CTSA	2.355892	0.020293
GSTO1	2.360066	0.022339
C8B	2.165772	0.022548
CTTN	1.409565	0.024113
MAP1B	2.12676	0.024151
HLA-A	1.928769	0.024227
TXNDC17	1.440572	0.024317
CYCS	2.292216	0.024959
FLNB	2.479079	0.026144
HEL-248	1.728656	0.02712
RAN	1.73492	0.027289
GGH	-1.46179	0.027394
RRBP1	2.088982	0.027495
CTSZ	1.341783	0.027853
HPX	2.197157	0.028591
SERPINB2	2.050389	0.030067
CTHRC1	2.654414	0.030089
GDI1	1.874735	0.030107
MYL6	2.653095	0.030347
PSMB3	2.909456	0.030551
CEMIP	1.251391	0.031225
PRDX1	1.508151	0.03238
CAPZB	2.571109	0.032739
H3F3A	2.100563	0.032929
RPS5	-1.37439	0.033292
GOLM1	1.785586	0.033937
COL2A1	-2.834	0.034111
RSU1	1.665719	0.034243
IGHG1	2.380441	0.035565
APOB	1.651462	0.039649
ACTR3	2.294632	0.040018
PSAP	-1.38679	0.040032
DBI	1.288004	0.041649
EXT2	-1.22732	0.042881
TUBB4A	2.098419	0.042965

LAMB2	-2.63587	0.043262
NRP2	1.468992	0.04363
FKBP4	1.310613	0.046013
CD44	2.635731	0.046305
CST6	1.725026	0.047319
MARCKS	2.707279	0.04733
FSCN1	1.774569	0.047547
COL11A1	1.651534	0.048675
DYNLL1	1.874016	0.048716
PLS3	2.224719	0.048785
ACTN4	2.1624	0.049809

Supplementary Figure and Figure legends

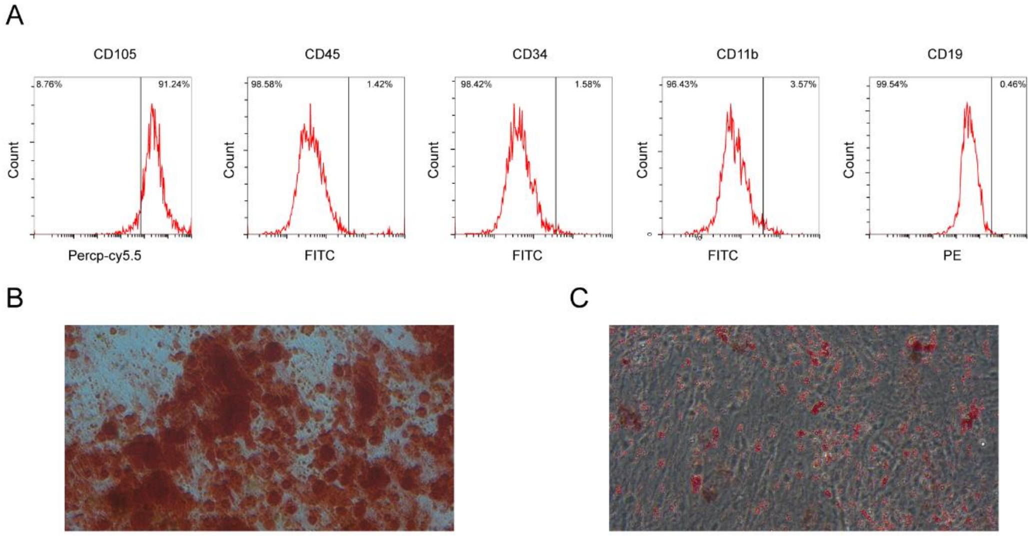

Figure S1. Identification of MSC by cell surface marker and differentiation.

A. Flow cytometry analysis of MSC surface markers, with positive markers being CD105 and negative markers being CD45, CD34, CD11b, and CD19. B. Identification of osteogenic differentiation of MSCs using Alizarin Red staining (magnification: ×200). C. Identification of adipogenic differentiation of MSCs using Oil Red O staining (magnification: ×200).

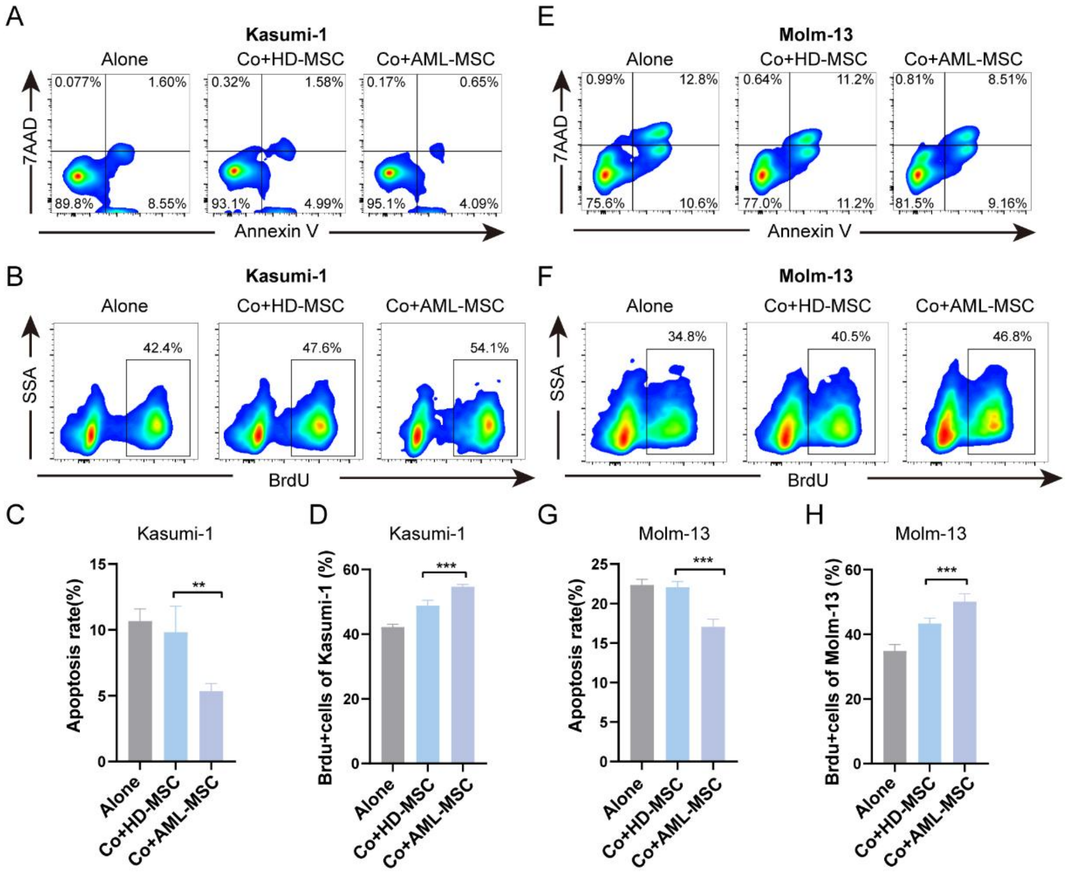

FigS2. AML-MSC promotes growth in leukemia cell lines compared to HD-MSC.

A-D, Analysis of apoptosis and BrdU assay in Kasumi-1 cells after 48 hours of co-culture with five cases each of AML-MSC and HD-MSC, including representative flow cytometry charts and statistical analysis (n=5). Alone, leukemia cells cultured alone; Co, leukemia cells cocultured with MSCs. E-H, Analysis of apoptosis and BrdU assay in Molm-13 cells after 48 hours of co-culture with five cases each of AML-MSC and HD-MSC, including representative flow cytometry charts and statistical analysis (n=5).

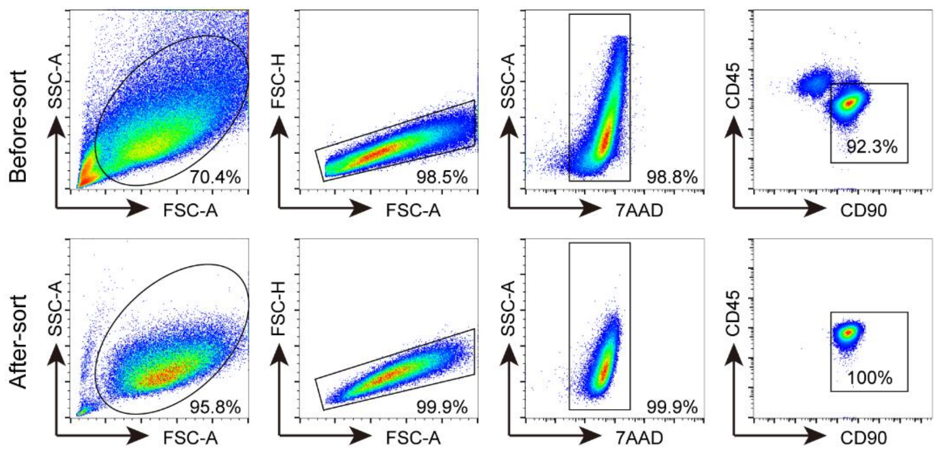

Figure S3. Gating strategy for FACS sorting of CD90+ MSCs.
After 48 hours of co-culturing MSCs from the knockdown and control groups with Kasumi-1 and Molm-13 cells, CD90+ MSCs were isolated using flow cytometric sorting.

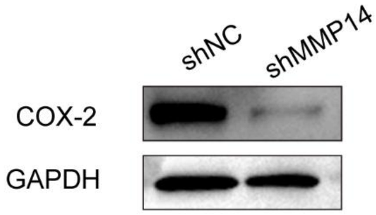

Figure S4 Inhibiting MMP14 in MSCs reduces COX-2 expression. Protein expression levels of COX-2 after MMP14 knockdown in MSC cells.

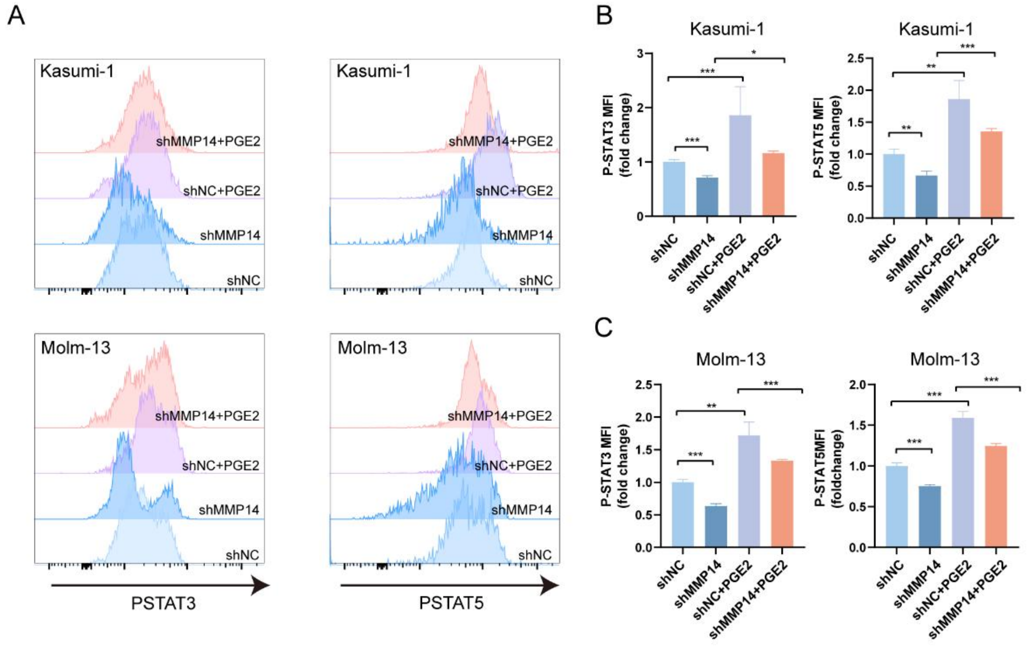

Figure S5. PGE2 exerts its effects via the JAK-STAT pathway.

A. Representative flow cytometry plots. Flow cytometric analysis was utilized to measure the levels of P-STAT3 and P-STAT5 proteins in Kasumi-1 and Molm-13 cells, either treated or untreated with PGE2 (1 μM), within the co-culture systems of knockdown and control groups. B-C. Statistical analysis of the levels of P-STAT3 and P-STAT5 proteins.

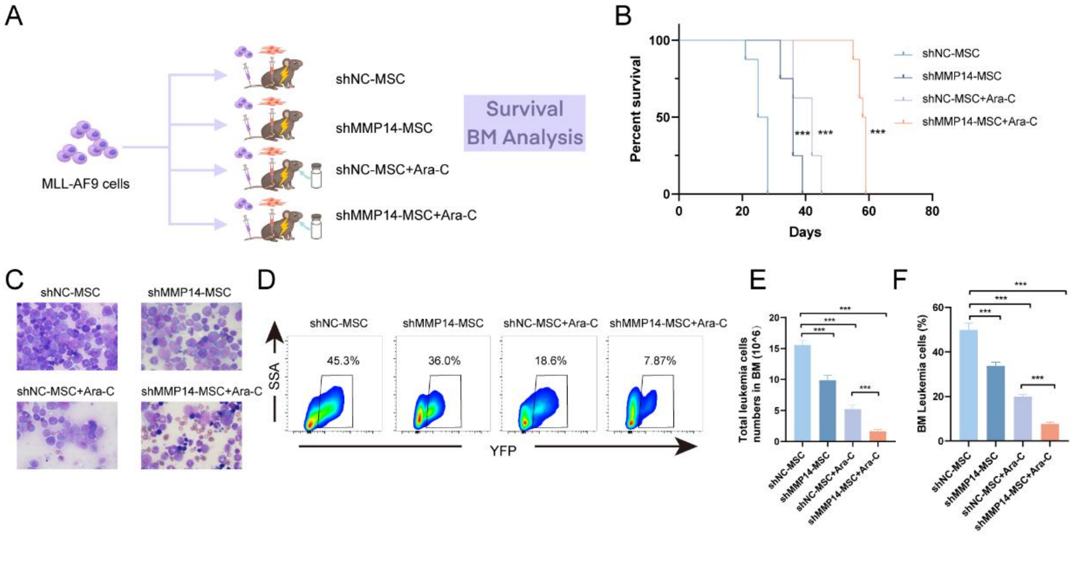

Figure S6. Inhibition of MSC-derived MMP14 enhances the cytotoxic effects of Ara-C in vivo

A. Overview of the experimental strategy. B. Survival analysis of mice (n=8). C. Representative images of Giemsa-Wright staining of BM cells from four groups of mice. D-F. Representative flow

cytometry plots of leukemia cells in mice, and statistical analysis of leukemia cell proportions and numbers.

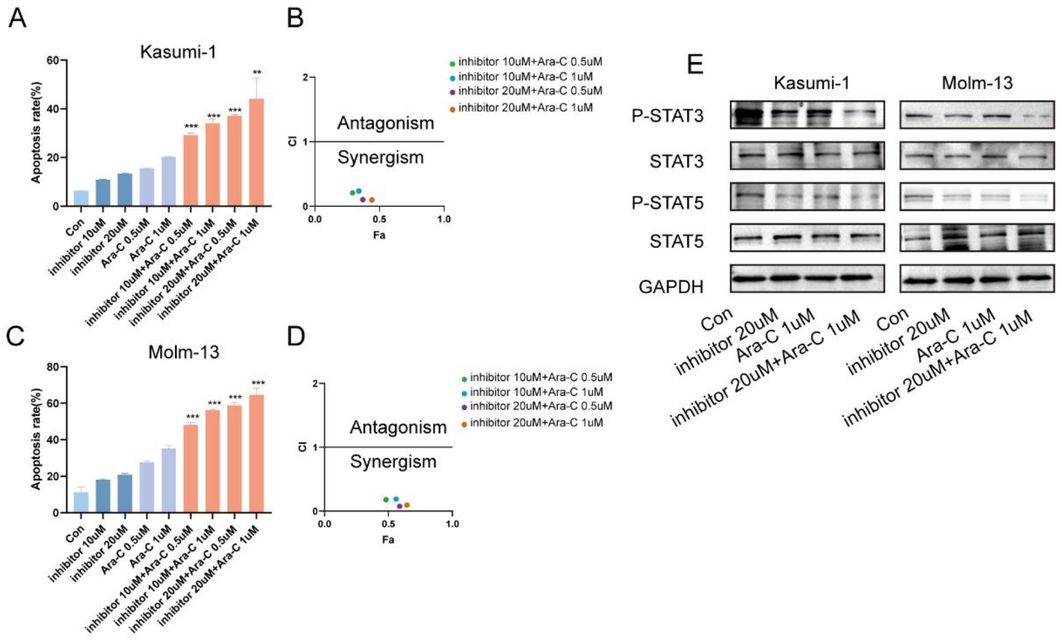

Fig. S7. MMP14 inhibitor NSC-405020 synergizes with Ara-C to induce apoptosis in AML cells. A-D. AML cell lines, Kasumi-1 and Molm-13, were co-cultured with MSCs and treated for 48 hours with the inhibitor, Ara-C, or their combination. Apoptosis rates were measured by flow cytometry, and the combination index (CI) was calculated using CompuSyn software, revealing synergistic effects (CI < 1) between the inhibitor and Ara-C. E. Western blot analysis of STAT3, P-STAT3, STAT5, and P-STAT5 protein expression in Kasumi-1 and Molm-13 cells co-cultured with MSCs.

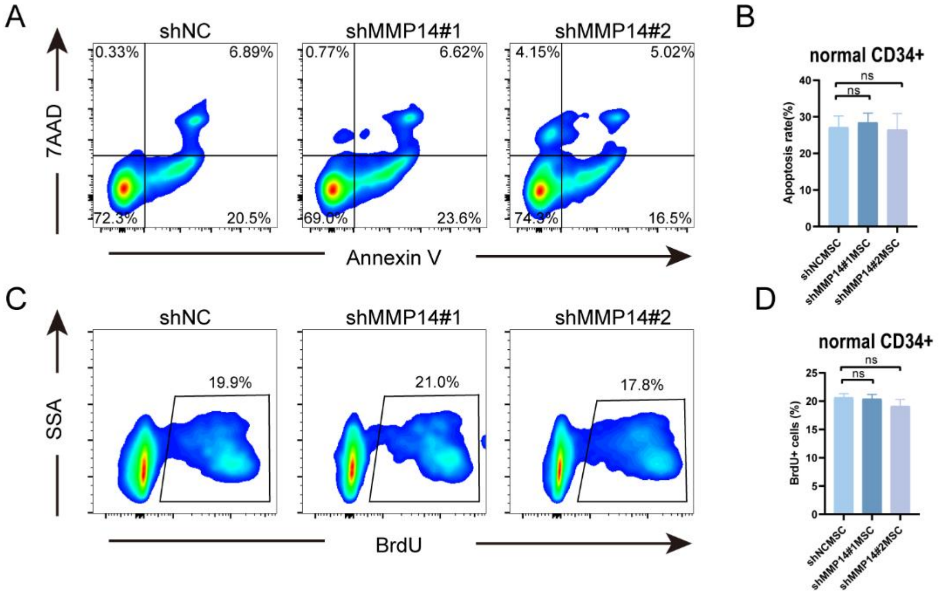

Fig. S8. Knockdown of MSC-derived MMP14 does not affect normal bone marrow-derived

CD34+ cells.

A-B. Normal bone marrow-derived CD34+ cells were co-cultured with MMP14 knockdown or control MSCs for 48 hours, and apoptosis rates of the CD34+ cells were assessed by flow cytometry (n=3). C-D. The proliferative capacity of normal CD34+ cells was measured using a BrdU incorporation assay.
